# Mitochondrial ROS drive resistance to chemotherapy and immune-killing in hypoxic non-small cell lung cancer

**DOI:** 10.1186/s13046-022-02447-6

**Published:** 2022-08-11

**Authors:** Iris C. Salaroglio, Dimas Carolina Belisario, Muhlis Akman, Sofia La Vecchia, Martina Godel, Dario Pasquale Anobile, Giacomo Ortone, Sabrina Digiovanni, Simona Fontana, Costanzo Costamagna, Menachem Rubinstein, Joanna Kopecka, Chiara Riganti

**Affiliations:** 1 Department of Oncology, University of Torino, via Santena 5/bis, 10126 Torino, Italy; 2 Department of Molecular Genetics, the Weizmann Institute of Science, Rehovot, Israel; 3 Interdepartmental Center of Research in Molecular Biotechnology, University of Torino, Torino, Italy

**Keywords:** Intermittent hypoxia, Chemo-immuno-resistance, CCAAT/enhancer binding protein-β, Mitochondrial ROS, Non-small cell lung cancer

## Abstract

**Background:**

Solid tumors subjected to intermittent hypoxia are characterized by resistance to chemotherapy and immune-killing by effector T-lymphocytes, particularly tumor-infiltrating Vγ9Vδ2 T-lymphocytes. The molecular circuitries determining this double resistance are not known.

**Methods:**

We analyzed a panel of 28 human non-small cell lung cancer (NSCLC) lines, using an in vitro system simulating continuous and intermittent hypoxia. Chemosensitivity to cisplatin and docetaxel was evaluated by chemiluminescence, ex vivo Vγ9Vδ2 T-lymphocyte expansion and immune-killing by flow cytometry. Targeted transcriptomics identified efflux transporters and nuclear factors involved in this chemo-immuno-resistance. The molecular mechanism linking Hypoxia-inducible factor-1α (HIF-1α), CCAAT/Enhancer Binding Protein-β (C/EBP-β) isoforms LAP and LIP, ABCB1, ABCC1 and ABCA1 transporters were evaluated by immunoblotting, RT-PCR, RNA-IP, ChIP. Oxidative phosphorylation, mitochondrial ATP, ROS, depolarization, O_2_ consumption were monitored by spectrophotometer and electronic sensors. The role of ROS/HIF-1α/LAP axis was validated in knocked-out or overexpressing cells, and in humanized (Hu-CD34^+^NSG) mice bearing LAP-overexpressing tumors. The clinical meaning of LAP was assessed in 60 NSCLC patients prospectively enrolled, treated with chemotherapy.

**Results:**

By up-regulating ABCB1 and ABCC1, and down-regulating ABCA1, intermittent hypoxia induced a stronger chemo-immuno-resistance than continuous hypoxia in NSCLC cells. Intermittent hypoxia impaired the electron transport chain and reduced O_2_ consumption, increasing mitochondrial ROS that favor the stabilization of C/EBP-β mRNA mediated by HIF-1α. HIF-1α/C/EBP-β mRNA binding increases the splicing of C/EBP-β toward the production of LAP isoform that transcriptionally induces ABCB1 and ABCC1, promoting the efflux of cisplatin and docetaxel. LAP also decreases ABCA1, limiting the efflux of isopentenyl pyrophosphate, i.e. the endogenous activator of Vγ9Vδ2 T-cells, and reducing the immune-killing. In NSCLC patients subjected to cisplatin-based chemotherapy, C/EBP-β LAP was abundant in hypoxic tumors and was associated with lower response to treatment and survival. LAP-overexpressing tumors in Hu-CD34^+^NSG mice recapitulated the patients’ chemo-immuno-resistant phenotype. Interestingly, the ROS scavenger mitoquinol chemo-immuno-sensitized immuno-xenografts, by disrupting the ROS/HIF-1α/LAP cascade.

**Conclusions:**

The impairment of mitochondrial metabolism induced by intermittent hypoxia increases the ROS-dependent stabilization of HIF-1α/LAP complex in NSCLC, producing chemo-immuno-resistance. Clinically used mitochondrial ROS scavengers may counteract such double resistance. Moreover, we suggest C/EBP-β LAP as a new predictive and prognostic factor in NSCLC patients.

**Supplementary Information:**

The online version contains supplementary material available at 10.1186/s13046-022-02447-6.

## Background

The alternation between the neo-angiogenesis and vessel collapse caused by tumor growth and extracellular matrix deposition generates repeated cycles of hypoxia and normoxia [1, 2]. This intermittent hypoxia generates tumor niches, with a partial O_2_ pressure (pO_2_) around 1–1.3%, resistant to chemotherapy, by multiple mechanisms. Hypoxic conditions subtract the hypoxia induced factor α subunit (HIF-α) from the O_2_-dependent hydroxylation by prolyl hydroxylase domain (PHD) enzymes and subsequent degradation [3], allowing the binding of α subunit with the O_2_-independent β subunit, and the transcriptional activation of more than 200 genes, promoting cell proliferation, epithelial mesenchymal transition, invasion, metabolic adaptation to the O_2_-deprived environment [1]. For instance, HIF-1α activates anti-apoptotic proteins of Bcl-2 family and survivins, destabilizes TP53, up-regulates DNA repair genes [4], reducing the efficacy of DNA damaging chemotherapeutic drugs, as cisplatin and gemcitabine [4, 5]. In parallel, the increased anaerobic metabolism of glucose, favored by the up-regulation of glucose transporter 1 (GLUT1) and glycolytic enzymes, determines intratumor acidosis, which neutralizes chemotherapeutic drugs that are weak bases, such anthracyclines, favoring their sequestration within lysosomes [6]. The reduction of oxidative phosphorylation (OXPHOS)-based metabolism and the increased mitophagy, which allows the recovery of ATP and oxide-reductive cofactors, further protect cancer cells from chemotherapy-induced damages [7]. Furthermore, HIF-1α induce the drug efflux ATP Binding Cassette (ABC) transporter B1/P-glycoprotein (ABCB1/Pgp) [8] and ABC transporter C1/multidrug resistance related protein 1 (ABCC1/MRP1) [9] that actively determine a multidrug resistant phenotype [10].

Hypoxia generates endoplasmic reticulum (ER) stress that produces cell death or survival, favoring or counteracting chemotherapy effects [11]. In neurons, intermittent hypoxia activates CCAAT/Enhancer Binding Protein (C/EBP) [12], which is of paramount importance in tumor chemoresistance. Indeed, by alternative splicing, C/EBP-β generates the full-length LAP isoform that up-regulates ABCB1, or the C-terminal LIP isoform that has opposite effects [13].

The ratio between LIP/LAP [14, 15] also affects the immuno-resistance in cancer, by pleiotropic mechanisms. First, ABCB1 impairs the immuno-sensitizing functions of calreticulin, an ER-residing protein exposed on plasma membrane upon ER stress or chemotherapy, triggering the phagocytosis by dendritic cells [16]. Second, LIP induces while LAP represses calreticulin [14]. Third, ER stress regulates the levels of ABC transporter A1 (ABCA1) [17] that we identified as the efflux transporter of isopentenyl pyrophosphate (IPP), an isoprenoid metabolite of the cholesterol synthesis [18] and an endogenous activator of Vγ9Vδ2 T-cells [19]. These T-cells are a subset with strong killing properties against tumor cells [20] and have been indicated as the immune-infiltrating population with the highest positive prognostic value in lung cancer [21]. To close the circle linking chemo- and immuno-resistance, we found that in hypoxic 3D cultures ABCB1 and ABCC1 are up-regulated, while ABCA1 is down-regulated [15]. This phenotype makes cells resistant to chemotherapy and immune-killing.

In the present work we focused on non-small cell lung cancer (NSCLC), characterized by resistance to chemotherapy or immunotherapy in up to 30% patients [22] and we investigated how the responses to chemotherapy and immune-killing by Vγ9Vδ2 T-cells are affected by hypoxia, reproducing in vitro progressively more severe intermittent hypoxia. We identified mitochondrial reactive oxygen species (mtROS) as inducers and druggable targets of chemo-immuno-resistance.

## Methods

### Chemicals

Fetal bovine serum (FBS) and culture medium were from Invitrogen Life Technologies (Carlsbad, CA). Plasticware for cell cultures was from Falcon (Becton Dickinson, Franklin Lakes, NJ). The protein content was assessed with the BCA kit from Sigma Aldrich. (St. Louis, MO). Cis-diammineplatinum (II) dichloride, docetaxel, elesclomol were purchased by Sigma Aldrich. [10-(2,5-dihydroxy-3,4-dimethoxy-6,ethylphenyl)decyl]triphenyl-phosphonium, monomethanesulfonate (mitoquinol or mitoQ) was from Cayman Chemical (Ann Arbor, MI).

### Cells

Human NSCLC cells (NCI-H1650, NCI-H1385, NCI-H460, NCI-H522, NCI-H661, NCI-H2126, NCI-H23, NCI-H1703, NCI-H1435, NCI-H596, NCI-H2286, NCI-H1437, NCI-H1651, NCI-H2085, NCI-H2342, NCI-H2073, NCI-H1793, NCI-H2170, NCI-H1299, NCI-H2066, NCI-H2347, NCI-H1734, NCI-H1563, NCI-H441, NCI-H1975, A549, Calu-3, NCI-H2228) were purchased from ATCC (Manassas, VA). Cells were maintained in the respective medium supplemented with 10% v/v FBS, 1% v/v penicillin-streptomycin, 1% v/v L-glutamine, in a Heracell incubator (ThermoFisher, Waltham, MA) with different pO_2_. The experimental conditions were set as it followed: 24 h at 20% O_2_ (normoxia), 24 h at 1% O_2_ (hypoxia), 12 h at 1% O_2_ followed by 12 h at 20% O_2_ (hypoxia/normoxia), 12 h at 1% O_2_ followed by 12 h at 20% O_2_ and 12 h at 1% O_2_ (hypoxia/normoxia/hypoxia or intermittent hypoxia).

### Cell viability

After the growth in normoxic or hypoxic conditions indicated, cells were incubated 48 h in normoxia (20% O_2_), with increasing concentrations (from 1 × 10^− 9^ to 1 × 10^− 5^ M) of cisplatin or docetaxel. Cell viability was assessed by the ATPlite Luminescence Assay System (PerkinElmer, Waltham, MA), as per manufacturer’s instructions, using a Synergy HT Multi-Detection Microplate Reader (Bio-Tek Instruments, Winooski, VT) to measure the relative luminescence units (RLUs). The RLUs of untreated cells were considered as 100% viability; the results were expressed as a percentage of viable cells versus untreated cells. The IC_50_ was calculated applying the [inhibitor] vs. normalized dose-response equation (GraphPad Prism 9 software, https://www.graphpad.com/).

### Flow cytometry

1 × 10^4^ cells were washed in phosphate-saline buffer (PBS), pH 7.2, 0.5% bovine serum albumin (BSA) and 2 mM EDTA, centrifuged at 300×g for 10 min, incubated 20 min at room temperature in the dark with 250 μl of Inside Fix reagent (Inside Stain Kit, Miltenyi Biotec., Bergisch Gladbach, Germany), centrifuged at 300×g for 5 minutes, washed with 1 ml of Inside Perm (Inside Stain Kit), centrifuged at 300×g for 5 minutes, and incubated 30 minutes at room temperature with the following antibodies (all from Miltenyi): anti-CD243/ABCB1 antibody (PE-Vio® 770-conjugated); anti-MRP1/ABCC1 antibody (PE-conjugated); anti-ABCA1(DyLight 488-conjugated). Cells were washed with 1 ml of Inside Perm reagent, centrifuged at 300×g for 5 minutes and read using a Guava® easyCyte flow cytometer (Millipore, Billerica, MA), equipped with the InCyte software (Millipore).

### PCR arrays and qRT-PCR

Total RNA was extracted and reverse-transcribed using the iScript™ cDNA Synthesis Kit (Bio-Rad Laboratories, Hercules, CA). The PCR arrays were performed on 1 μg cDNA, using customized Plus PCR Arrays (Bio-Rad Laboratories) pre-coated with primers for the ABC transporters or the transcription factors indicated in the Results section, as per manufacturer’s instructions. Data analysis was performed with the PrimePCR™ Analysis Software (Bio-Rad Laboratories). The qRT-PCR was performed with the IQ SYBR Green Supermix (Bio-Rad Laboratories). The primer sequences, designed using the qPrimerDepot database (http://primerdepot.nci.nih.gov/), were reported in the Additional File 1 (Supplemental Table S1). The relative quantification was performed by comparing each PCR product with the housekeeping PCR product *S14*, using the Bio-Rad Software Gene Expression Quantitation (Bio-Rad Laboratories).

### Promoter and transcription factors analysis

The promoter sequences were identified from the Eukariotic Promoter Database (EPD; https://epd.epfl.ch//index.php), using as inputs: https://epd.epfl.ch/cgi-bin/get_doc?db=hgEpdNew&format=genome&entry=ABCB1_1 (RefSeq NM_001348945) for *ABCB1*, https://epd.epfl.ch/search_EPDnew.php?query=ABCC1&db=human (RefSeq NM_004996) for *ABCC1,* https://epd.epfl.ch/cgi-bin/get_doc?db=hgEpdNew&format=genome&entry=ABCA1_1 (RefSeq NM_080282) for *ABCA1*. The transcription factors predicted to bind *ABCB1*, *ABCC1* and *ABCA1* promoters (Additional File 1; Supplemental Table S2) were identified using the TRANSFAC software v 8.3 (http://alggen.lsi.upc.es/cgi-bin/promo_v3/promo/promoinit.cgi?dirDB=TF_8.3) [23].

### Immunoblotting

Cells were lysed in the MLB buffer (125 mM Tris-HCl, 750 mM NaCl, 1%v/v NP40, 10%v/v glycerol, 50 mM MgCl_2_, 5 mM EDTA, pH 7.5, supplemented with the protease inhibitor cocktail set III (Sigma Aldrich), 25 mM NaF, 1 mM NaVO_4_, 10 mg/ml aprotinin), sonicated and centrifuged at 13000×g for 10 minutes at 4 °C. 50 μg of proteins were subjected to immunoblotting and probed with the following antibodies: anti-HIF-1α (clone 54, BD Transduction Laboratories), C/EBP-β (clone C-19, directed against the common C-terminus of LIP and LAP, Santa Cruz Biotechnology Inc., Santa Cruz, CA), followed by a peroxidase-conjugated secondary antibody. Anti-actin (clone C-4, Sigma Aldrich) was used as a control of equal protein loading. The proteins were detected by enhanced chemiluminescence (Bio-Rad Laboratories). In co-immunoprecipitation assays, 100 μg of whole cell lysates were immuno-precipitated at 4 °C overnight with the PureProteome Protein A/G Mix Magnetic Beads (Millipore) as per manufacturer’s instruction, in the presence of the anti-C/EBP-β antibody (clone C-19, diluted 1/50), then blotted with the anti-HIF-1α antibody as reported above.

### RNA immunoprecipitation (RNA-IP)

Total RNA was isolated using the Magna RIP™ RNA-Binding Protein Immunoprecipitation Kit (Sigma Aldrich), as per manufacturer’s instructions. 2 μg RNA were immuno-precipitated for 3 h at 4 °C using an anti-HIF-1α antibody (PA1–16601, Invitrogen Life Technologies, Milan, Italy). A blank was prepared by incubating the samples without the antibody. The immunoprecipitated RNA was retro-transcribed using the iScript™ cDNA Synthesis Kit (Bio-Rad Laboratories). RT-PCR was performed with the IQ SYBR Green Supermix (Bio-Rad Laboratories), using primers upstream and downstream the hypoxia-response elements (HREs) contained in C/EBP-β LAP and LIP, respectively (Additional File 1; Supplemental Table S1). The expression in normoxic cells was considered as 1. The levels of LAP and LIP mRNA in the other experimental condition were expressed as fold-enrichment versus normoxic cells, using the Bio-Rad Software Gene Expression Quantitation (Bio-Rad Laboratories).

### Chromatin immunoprecipitation (ChIP)

Cells were lysed and sonicated as reported previously [18]. 200 μl of samples were used as inputs. The remaining lysates were pretreated for 2 h at 4 °C with the Magna ChIP™ Protein A + G Magnetic Beads (Sigma Aldrich), as per manufacturer’s instructions. Samples were incubated overnight with the anti-C/EBP-β antibody (clone C-19, Santa Cruz Biotechnology Inc.) or without antibody, as a blank. The recovered DNA was washed, eluted with the elution buffer (0.1 M NaHCO_3_, 1% w/v SDS), heated at 65 °C for 6 h and incubated with proteinase K for 1 h at 55 °C. Samples were cleaned by Qiaquick spin columns (Qiagen, Venlo, The Netherlands). The CAAT sites on *ABCB1, ABCC1* and *ABCA1* promoters were identified using the Transfac® Database (http://genexplain.com/transfac/). Inputs and immunoprecipitated samples were analysed by RT-PCR, using primers designed to amplify the sequence around the CAAT sites in *ABCB1, ABCC1* and *ABCA1* promoters, as well as nonspecific primers, used as negative internal controls (Additional File 1; Supplemental Table S1).

### Cell transfection

1 μg pcDNA4/TO expression vectors (Invitrogen Life Technologies) for C/EBP-β LAP and LIP, produced as reported previously [24], were co-transduced with 1 μg pcDNA6/TR vector (Invitrogen Life Technologies) in 1 × 10^6^ cells. Stable TetON clones were generated by selecting cells with 2 μg/ml blasticidin S (ThermoFisher) and 100 μg/ml zeocin (InvivoGen, San Diego, CA). LAP and LIP induction was activated by adding 1 μg/ml doxycycline (Sigma Aldrich) in the culture medium. LAP and LIP expression was analyzed by immunoblotting 24 h after doxycycline treatment. For silencing, 1 × 10^6^ cells were transduced with 1 μg of a green fluorescence protein (GFP)-lentiviral plasmid containing a non-effective 29-mer scrambled shRNA cassette (Origene, Rockville, MD), two different sequences targeting HIF-1α (TL320380, Origene), two different sequences targeting C/EBP-β (TL320301, Origene). Stably silenced clones were generated by selecting cells with 0.25 μg/ml puromycin (InvivoGen) for 4 weeks. The silencing was verified by RT-PCR and immunoblotting.

### Release of IPP

The efflux of IPP was measured by radiolabelling 1 × 10^6^ cells for 1 h with 0.02 mCi of [^14^C]-IPP (50 mCi/mmol; Amersham International, Piscataway, NJ), extracting lipids, isolating the IPP by thin layer chromatography and counting radiolabelled IPP by liquid scintillation [18]. Results were expressed as nanomoles/ml, according to the relative calibration curve.

### Vγ9Vδ2 T-lymphocytes activation and tumor killing

Blood samples were obtained from healthy blood donors (Blood Bank of the AOU Città della Salute e della Scienza, Torino, Italy; DG 767/2015). After isolation on a Ficoll-Hypaque density gradient, peripheral blood mononuclear cells (PBMC) were sorted using the TCRγ/δ^+^T Cell Isolation Kit (Miltenyi Biotec.). The presence of Vγ9Vδ2 T-lymphocytes was confirmed by staining 5 × 10^5^ isolated cells with anti-TCR Vγ9 (VioBlue conjugated; Miltenyi Biotec.) and anti-CD3 (fluorescein-isothiocyanate - FITC - conjugated, Miltenyi Biotec) antibodies. Samples with > 80% Vγ9^+^/CD3^+^ cells were incubated 48 h with 1 μM zoledronic acid (Sigma Aldrich) and 10 IU/ml IL-2 (Sigma Aldrich), to expand Vγ9Vδ2 T-lymphocytes [19], then 5 × 10^5^ Vγ9Vδ2 T-lymphocytes were cultured overnight with NSCLC cells at 1:1 ratio. After this incubation period, the amount of active and proliferating Vγ9Vδ2 T-lymphocytes was measured by staining cells present in the supernatants with anti-Ki67 (FITC-conjugated) and anti-INF-γ (allophycocyanin - APC - conjugated) antibodies (Miltenyi Biotec.), and quantified with a Guava® easyCyte flow cytometer (InCyte software). Results were expressed as percentage of Vγ9^+^Ki67^+^IFNγ^+^over Vγ9^+^cells. Vγ9Vδ2 T-lymphocytes killing was measured as reported [15]. After Vγ9Vδ2 T-lymphocytes/NSCLC cells co-incubation, adherent (i.e. tumor cells) were washed twice with PBS, detached by gentle scraping and stained with the Annexin V/Propidium Iodide kit (Sigma Aldrich.), as per manufacturer’s instruction. The fluorescence was acquired using a Guava® easyCyte flow cytometer (InCyte software). The percentage of Annexin V^+^/Propidium Iodide^+^ cancer cells was considered as an index of Vγ9Vδ2 T-lymphocytes killing.

### Electron transport chain (ETC) activity

Mitochondria were isolated from 10 × 10^6^ cells, lysed in 0.5 ml lysis buffer (5 mM Tris-HCl, 100 mM KCl, 5 mM MgCl_2_, 1.8 mM ATP, 1 mM EDTA, pH 7.2), supplemented with Protease Inhibitor Cocktail III, 1 mM phenylmethylsulfonyl fluoride and 250 mM NaF. Samples were centrifuged at 650×g for 3 minutes at 4 °C, the supernatants were re-centrifuged at 13000×g for 5 minutes at 4 °C. The pellets, containing mitochondria, washed with lysis buffer, were resuspended in 0.25 ml resuspension buffer (250 mM sucrose, 15 mM K_2_HPO_4_, 2 mM MgCl_2_, 0.5 mM EDTA). 50 μl aliquots were sonicated and used for the measurement of protein content. 10 μg of each sonicated sample were analyzed by SDS-PAGE and immunoblotting with an anti-porin antibody (clone 20B12AF2, Abcam, Cambridge, UK) to confirm the presence of mitochondrial proteins in the extracts. The electron efflux from complex I to complex III, taken as an index of the mitochondrial respiratory activity [25], was measured on 50 μg of non-sonicated mitochondrial samples, re-suspended in 0.2 ml of buffer A (5 mM KH_2_PO_4_, 5 mM MgCl_2_, 5%w/v BSA; pH 7.2) and 0.1 ml of buffer B (25%w/v saponin, 50 mM KH_2_PO_4_, 5 mM MgCl_2_, 5%w/v BSA, 0.12 mM oxidized cytochrome c, 0.2 mM NaN_3_, which blocks complex IV allowing the accumulation of reduced cytochrome c; pH 7.5). The reaction mix was allowed to equilibrate for 5 minutes at room temperature. The cytochrome c reduction reaction was monitored for 5 minutes after adding 0.15 mM NADH, reading the absorbance changes at 550 nm by a Synergy HT Multi-Detection Microplate Reader (Bio-Tek Instruments). Results were expressed as nanomoles of reduced cytochrome c /min/mg mitochondrial proteins.

### O_2_ consumption rate (OCR)

25 × 10^4^ cells were seeded in 96-well microplates (Nunc, Rochester, NY). After 24 h, the Resipher oxygen sensing lid (Lucid Scientific, Atlanta, MA) was positioned upon the plate [26]. Cells were subjected to these culture conditions: 36 h in normoxia, 36 h in hypoxia, 18 h in hypoxia followed by 18 h normoxia, 12 h hypoxia followed by 12 h normoxia and 12 h hypoxia, to monitor the O_2_ consumption over the same period. Live OCR was monitored continuously for 36 h by measuring the flux of O_2_ diffusing into the cells from the air above the well. The measurement was performed by sensing the O_2_ concentration gradient across a range of heights throughout the media and then calculating the flux of O_2_, according to Fick’s first and second laws [27]. Data were analyzed using the Resipher web application (Lucid Scientific).

### Mitochondrial ATP

ATP levels in mitochondrial extracts were measured with the ATP Bioluminescent Assay Kit (FLAA; Sigma Aldrich), as per manufacturer’s instructions. Results were expressed as nanomoles/mg mitochondrial proteins.

### Mitochondrial depolarization

1 × 10^6^ cells were washed with PBS, detached by gentle scraping and incubated for 30 minutes at 37 °C with 2 μM of the fluorescent probe JC-1 (Biotium Inc., Hayward, CA), centrifuged at 13000×g for 5 minutes and re-suspended in 0.5 ml PBS. The red fluorescence (λ excitation: 550 nm, λ emission: 600 nm), index of polarized mitochondria, and the green fluorescence (λ excitation: 485 nm; λ emission: 535 nm), index of depolarized mitochondria fluorescence, were read using a Synergy HT Multi-Detection Microplate Reader (Bio-Tek Instruments). The relative fluorescence units (RFUs) were used to calculate the percentage of green (depolarized)/red (polarized) mitochondria, considered an index of damaged mitochondria [28].

### Mitochondrial permeability transition pore (mPTP) activity

The opening of the mPTP, a second index of mitochondria depolarization and damage, was measured with the Mitochondrial Permeability Transition Pore Assay Kit (BioVision, Milpitas, CA), as per manufacturer’s instructions, using a Guava EasyCyte flowcytometer (Millipore), equipped with the InCyte software (Millipore). 1 × 10^5^ unstained cells were used to set the threshold of autofluorescence and subtracted from the stained cells. Results were expressed as percentage of fluorescent cells over total cells.

### Total and mitochondrial ROS

10 × 10^6^ cells were washed with PBS and detached by gentle scraping. One 50 μl aliquot was sonicated and used to measure cellular proteins. The remaining cells were treated for 30 min at 37 °C with 5 μM of the ROS-sensitive fluorescent probes 5-(and-6)-chloromethyl-2′,7′-dichlorodihydro-fluorescein diacetate (CM-H_2_DCFDA) (ThermoFisher) or with 5 μM MitoSOX (ThermoFisher), to measure total and mtROS, respectively. The RFUs were converted into nanomoles ROS/mg proteins, according to a titration curve performed with serial dilutions of H_2_O_2_.

### Antioxidant enzymes activity

The activity of cytosolic superoxide dismutase 1 (SOD1) and mitochondrial superoxide dismutase 2 (SOD2) was measured on 10 μg proteins of the respective extracts, obtained as indicated above, in 100 μl PBS containing 50 μM xanthine, 5 U/ml xanthine oxidase, 1 μg/ml oxidized cytochrome c. The rate of cytochrome c reduction, which is inhibited by SOD, was monitored for 5 minutes by reading the absorbance at 550 nm with a Synergy HT Multi-Detection Microplate Reader (Bio-Tek Instruments). Results were expressed as μmoles reduced cytochrome c/min/mg cytosolic or mitochondrial proteins [29]. Catalase and glutathione peroxidase (GPX) were measured on whole cell lysates using the Catalase Activity Assay Kit (Colorimetric/Fluorometric) (Abcam) and the Glutathione Peroxidase Assay Kit (Colorimetric) (Abcam), as per manufacturer’s instructions. The absorbance was converted into nmoles/min/mg proteins, according to the titration curves of the kits.

### Reduced glutathione (GSH) and oxidized glutathione (GSSG) measurement

1 × 10^6^ cells were rinsed with 480 μl PBS plus 120 μl of 6.5% w/v 5-sulfosalicylic acid to precipitate proteins, incubated at 4 °C for 1 h and centrifuged for 13,000×g for 5 minutes at 4 °C. Total (GSH + GSSG) glutathione was measured in 20 μl of the lysate by adding 20μlofstock buffer (143 mM NaH_2_PO_4_ and 63 mM EDTA, pH 7.4), 200 μl of daily reagent (10 M 5,5’dithiobis-2-nitrobenzoic acid and 2 mM NADPH, diluted in stock buffer), 40 μl glutathione reductase (8.5 U/mL). To measure oxidized glutathione (GSSG), 10 μl of 2-vinylpyridine were added to 200 μl of cell lysate for 1 h, to derivatize GSH. The remaining GSSG was measured on 40 μl of lysates, as described above. The reaction kinetics was followed for 5 minutes, reading the absorbance at 415 nm with a Synergy HT Multi-Detection Microplate Reader. Total glutathione (GSH + GSSG) and oxidized glutathione (GSSG) were expressed as pmoles glutathione/min/mg cellular proteins. GSH was obtained by subtracting GSSG values from (GSH + GSSG) values [29]. Results were then expressed as GSH/GSSG ratio.

### Patient enrolment and immunohistochemical analyses

60 patients with confirmed histological diagnosis of NSCLC, candidate to receive cisplatin/carboplatin as first-line therapy, were prospectively enrolled at San Lugi Gonzaga Hospital, Orbassano, and Città della Salute e della Scienza Hospital, Torino, Department of Oncology, University of Torino, Italy (March 2018–January 2020). Each patient was anonymized and indicated as “unknown patient number” (UPN). The pathological features, the smoking habits, the clinical follow-up (progression free survival, PFS; overall survival, OS) of patients, performed at the Thoracic Oncology Unit, San Luigi Gonzaga Hospital, are reported in the Additional File 1 (Supplemental Table S3). Formalin-fixed paraffin-embedded (FFPE) samples of patients were analyzed for the presence of hypoxic areas using the pimonidazole-based Hypoxyprobe™ Kit (Hypoxyprobe Inc., Burlington, MA), according to the manufacturer’s instructions. The same sections were also stained with an antibody recognizing only the N-terminal portion of C/EBP-β, corresponding to LAP (clone 21A1, ThermoFisher), followed by peroxidase-conjugated horseradish antibody (Dako, Glostrup, Denmark). Nuclei were counterstained with hematoxylin (Sigma Aldrich). C/EBP-β LAP was considered positive when a weak-to strong nuclear or cytosolic positivity was shown. The tumor proportion positivity was recorded. Patients were divided into LAP^*low*^ and LAP^*high*^, if the tumor proportion of LAP staining was respectively below or equal/above the median value. The Ethics Committee of San Luigi Gonzaga Hospital, Orbassano, Italy approved the study (#73/2018).

### Immuno-xenografts

1 × 10^6^ NCI-H2228 C/EBP-β LAP-overexpressing cells, mixed with 100 μl Matrigel (Sigma Aldrich), were injected subcutaneously (s.c.) in female NOD SCID-γ (NSG) mice engrafted with human hematopoietic CD34^+^ cells (Hu-CD34^+^; The Jackson Laboratories, Bar Harbor, MA). Mice were housed (5 per cage) under 12 h light/dark cycle, with food and drinking provided ad libitum. When indicated, doxycycline (1 mg/ml) was added daily to the drinking water to induce C/EBP-β LAP intratumorally. Tumor growth was measured daily by caliper, according to the equation (LxW^2^)/2, where L = tumor length and W = tumor width. In a preliminary experimental set, when tumors reached the volume of 50mm^3^, animals (4/group) were randomized and treated for 6 weeks as it follows: vehicle group, treated with 0.1 ml saline solution intravenously (i.v.), once a week; cisplatin group, treated with 2 mg/kg cisplatin i.v., once a week; mitoquinol (mitoQ) groups, treated with 10, 25, 50, 100, 200 mg/kg daily via oral gavage; cisplatin+mitoQ groups, treated with 2 mg/kg cisplatin i.v., once a week, and 10, 25, 50, 100, 200 mg/kg daily via oral gavage. In a second experimental set, when tumors reached the volume of 50mm^3^, animals (6/group) were randomized and treated for 6 weeks as it follows: vehicle group, treated with 0.1 ml saline solution i.v., once a week; cisplatin group, treated with 2 mg/kg cisplatin i.v., once a week; mitoQ group, treated with 100 mg/kg daily via oral gavage; cisplatin+mitoQ group, treated with 2 mg/kg cisplatin i.v., once a week, and 100 mg/kg mitoQ daily via oral gavage. In both experimental sets, tumor volumes were monitored by caliper and animals were euthanized at day 49 after randomization with zolazepam (0.2 ml/kg) and xylazine (16 mg/kg). Animal weights were monitored throughout the study. Tumors were excised, weighted, and photographed. Tumor sections, fixed in 4% v/v paraformaldehyde, were stained with hematoxylin/eosin (Sigma Aldrich) or immunostained for: Hypoxyprobe™ Kit, LAP (clone 21A1, ThermoFisher), C/EBP-β LAP (ThermoFisher), ABCB1 (Novus Biologicals, Centennial, CO), ABCC1 (MyBioSource, San Diego, CA), ABCA1 (Abcam), cleaved (Asp175) caspase-3 (Cell Signaling Technology, Danvers, MA), followed by a peroxidase-conjugated secondary antibody (Dako). Nuclei were counterstained with hematoxylin (Sigma Aldrich). Sections were examined with a Leica DC100 microscope. To evaluate the intratumour Vγ9δ2 T-lymphocytes, the tumors were digested with 1 mg/ml collagenase (Sigma Aldrich) and 0.2 mg/ml hyaluronidase (Sigma Aldrich) for 1 h at 37 °C and filtered using a 70 μm cell strainer to obtain a single cell suspension. Infiltrating immune cells were collected by centrifugation on Ficoll-Hypaque density gradient and immunostained with the following antibodies (Miltenyi Biotec): anti-CD3, anti-Vγ9, anti-Ki67, anti-INF-γ, as indicated in the “Vγ9Vδ2 T-lymphocytes activation” section. Cells were quantified with using Guava® easyCyte flow cytometer and InCyte software. Results were expressed as percentage of Vγ9^+^Ki67^+^IFNγ^+^ over CD3^+^ cells. At 3.5 weeks and immediately after the euthanasia, 200 μl blood were collected to measure the following parameters: red blood cells (RBC), white blood cells (WBC), haemoglobin (Hb), platelets (PLT), as indexes of bone marrow function; lactate dehydrogenase (LDH), aspartate aminotransferase (AST), alanine aminotransferase (ALT), alkaline phosphatase (AP), as indexes of liver function; creatinine, as index of kidney function; creatine phosphokinase (CPK), as index of muscle/heart damage, using commercially available kits from Beckman Coulter Inc. (Miami, FL). Animal care and experimental procedures were approved by the Italian Ministry of Health (#627/2018-PR, 10/08/2018).

### Statistical analysis

All data in the text and figures are provided as means ± SD. The results were analyzed by a one-way analysis of variance (ANOVA), using Statistical Package for Social Science (SPSS) software (IBM SPSS Statistics v.19). *p* < 0.05 was considered significant. The Kaplan-Meier method was used to calculate the PFS (survival from the beginning of chemotherapy to the first sign of disease’s progression) and OS (survival from the beginning of chemotherapy until patients’ death). Log rank test was used to compare the outcome of LAP^*low*^ and LAP^*high*^ groups. The patient and animal sample sizes were calculated with the G*Power software (www.gpower.hhu.de), setting α < 0.05 and 1-β = 0.80. Researchers analyzing the results were unaware of the treatments received.

## Results

### Intermittent hypoxia up-regulates ABCB1 and ABCC1, inducing chemoresistance in non-small cell lung cancer cells

**Fig. 1:**
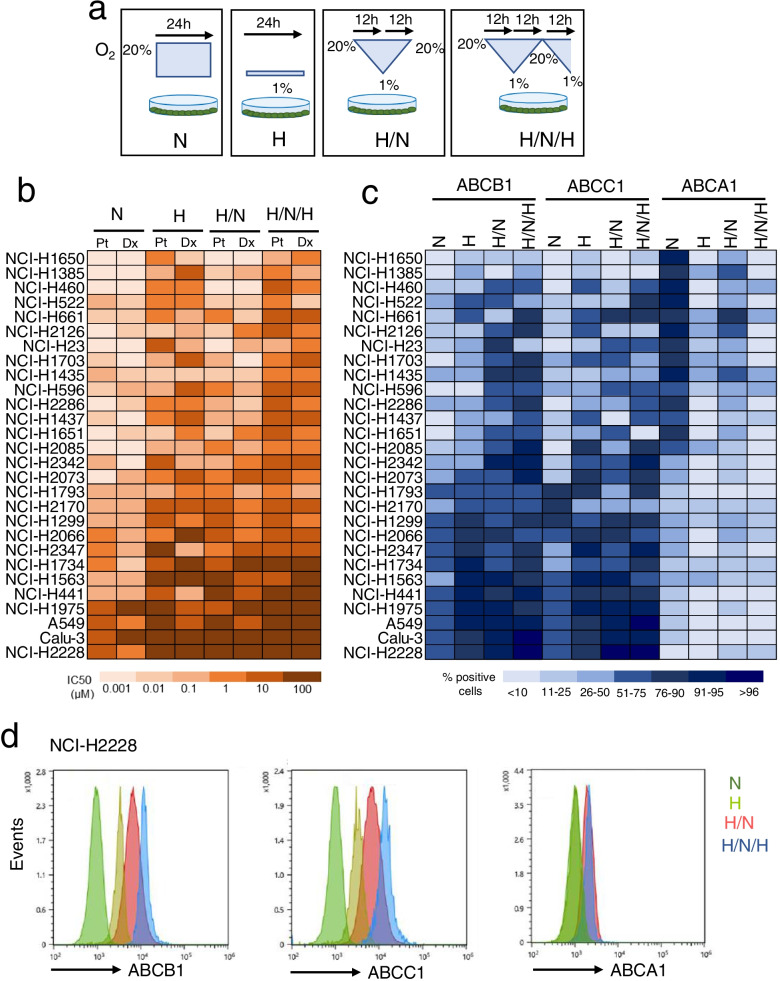
Intermittent hypoxia induces chemo- and immuno-resistance in non-small cell lung cancer cells. **a** Experimental settings. Cells were cultured in the following conditions: normoxia (at 20% O_2_ for 24 h, N), hypoxia (at 1% O_2_ for 24 h, H), hypoxia/normoxia (12 h at 1% O_2_ followed by 12 h at 20% O_2_, H/N), hypoxia/normoxia/hypoxia or intermittent hypoxia (12 h at 1% O_2_ followed by 12 h at 20% O_2_ and 12 h at 1% O_2_, H/N/H). **b** 28 non-small cell lung cancer cell lines were incubated as reported in **a**, then subjected to a 48 h treatment in normoxia (20% O_2_) with increasing concentrations (from 1 × 10^− 9^ to 1 × 10^− 5^ M) of cisplatin (Pt) and docetaxel (Dx). Cell viability was measured by a chemiluminescence-based assay, in technical quadruplicates (*n* = 3 biological replicates). The IC_50_ was calculated with the GraphPad Prism 9 software and is represented in a colorimetric scale. **c** 28 non-small cell lung cancer cell lines were incubated as reported in **a**, then subjected to flow cytometry analysis to evaluate the levels of ABCB1, ABCC1 and ABCA1, in technical duplicates (*n* = 3 biological replicates). The percentage of positive cells is represented in a colorimetric scale. **d** Representative histograms of ABCB1, ABCC1 and ABCA1 levels, measured by flow cytometry, in NCI-H2228 cells

To compare the effects of normoxia and progressively more severe hypoxia, we grew 28 human NSCLC cell lines in normoxia (24 h incubation at 20% O_2_), hypoxia (24 h incubation at 1% O_2_), hypoxia/normoxia (12 h at 1% O_2_, followed by 12 h at 20% O_2_), hypoxia/normoxia/hypoxia, or intermittent hypoxia (12 h at 1% O_2_, followed by 12 h at 20% O_2_ and 12 h at 1% O_2_) (Fig. 1a). Notwithstanding the different drug sensitivity among each cell line, the IC_50_ cisplatin and docetaxel, measured after a 48 h growth in normoxia, progressively increased moving from cells previously cultured in normoxia to cells previously cultured in continuous hypoxia, one shot of hypoxia/normoxia or intermittent hypoxia (Fig. 1b). Plasma-membrane associated ABCB1 and ABCC1, two transporters effluxing cisplatin and docetaxel [10], increased progressively in cells subjected to hypoxia, hypoxia/reoxygenation and intermittent hypoxia, compared to their normoxic counterparts (Fig. 1c). In line with other chemoresistant tumors [15], high levels of ABCB1 and ABCC1 were paralleled by low levels of ABCA1 in normoxia and – at greater extent – in hypoxia, hypoxia/normoxia and intermittent hypoxia (Fig. 1c). To investigate the mechanisms underlying, we next focused on the strongly resistant NCI-H2228 cells, which had one of the highest IC_50_ values of cisplatin and docetaxel (Fig. 1b; Additional file 1: Supplemental Fig. S1a-b), and highest ratio between ABCB1/ABCB1 versus ABCA1 (Fig. 1c-d) even in normoxia, further increased in all the hypoxic settings. In parallel, we performed key experiments NCI-H1563 cells, a sensitive cell line characterized by low IC_50_ of cisplatin and docetaxel, low expression of ABCB1/ABCC1 and high expression of ABCA1 in normoxia. This chemo-immuno-sensitive phenotype was driven toward a chemo-immuno-resistant phenotype (higher IC_50_, higher ABCB1/ABCC1 level, lower ABCA1 level) in hypoxia, particularly in intermittent hypoxia.

### Intermittent hypoxia increases HIF-1α-binding to C/EBP-β mRNA and the splicing of C/EBP-β into LAP isoform

**Fig. 2:**
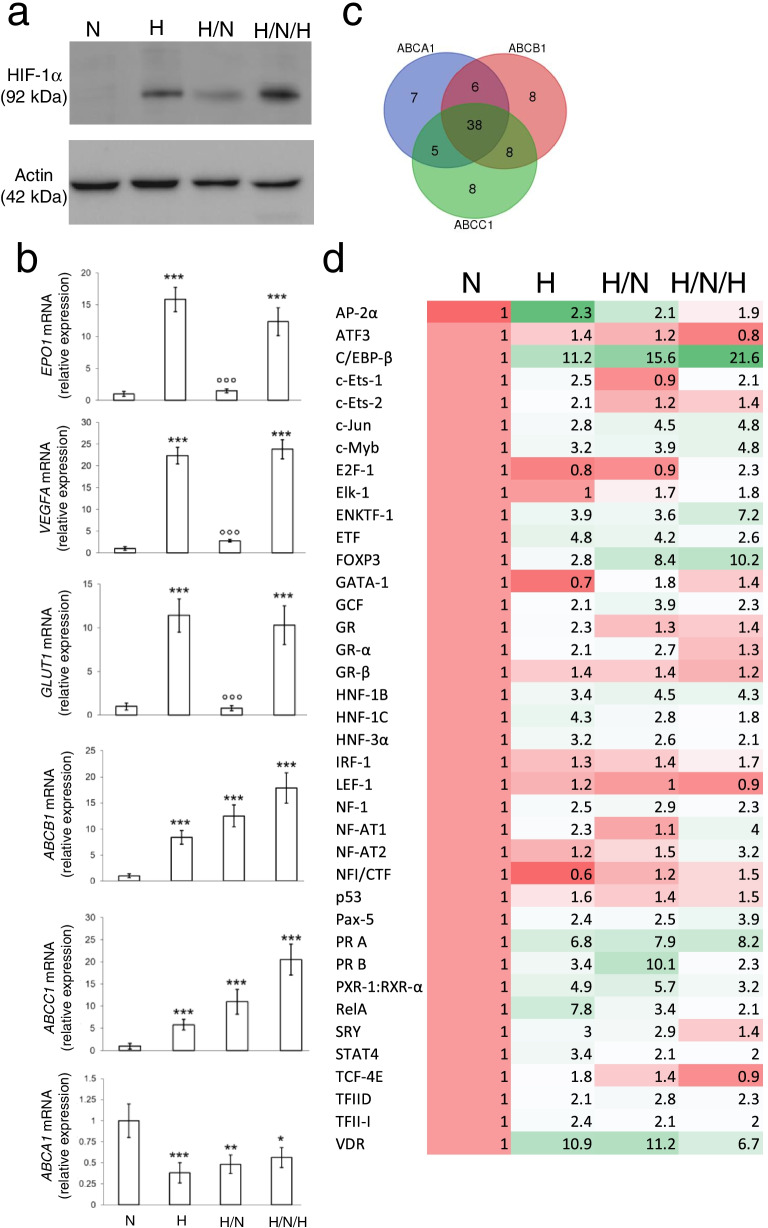
Intermittent hypoxia modulates ABCB1, ABCC1 and ABCA1 expression by changing C/EBP-β levels. NCI-H2228 cells were cultured as indicated in Fig. 1a. **a** Immunoblot of HIF-1α in whole cell extracts. Actin is included as control of equal protein loading. The image is representative of 1 out of 3 experiments. **b** *EPO1*, *VEGFA*, *GLUT1*, *ABCB1*, *ABCC1*, *ABCA1* mRNAs, measured by RT-PCR, in technical triplicates. Data are means±SD (*n* = 4 biological replicates). *p < 0.05,**p < 0.01,***p < 0.001: H, H/N, H/N/H versus N cells; °°°*p* < 0.001: H/N cells versus H cells. **c.** Venn diagram representing the transcription factors binding *ABCB1*, *ABCC1* and *ABCA1* promoters (TRANSFAC software). **d** The expression levels of 38 transcription factors commonly binding *ABCB1*, *ABCC1*, *ABCA1* promoters was measured by RT-PCR array and is represented in a colorimetric scale (*n* = 3 biological replicates). The expression of each transcription factor in normoxic cells was considered 1; the relative expression in the other experimental conditions is indicated in the heatmap

As expected, hypoxia increases the amount of HIF-1α protein, which was reduced by a hypoxia/normoxia cycle, and re-induced by intermittent hypoxia (Fig. 2a). Direct HIF-1α target genes, such as erythropoietin (*EPO1*), vascular endothelial growth factor A *(VEGFA)* and *GLUT1*, varied accordingly to HIF-1α proteins. By contrast, *ABCB1* and *ABCC1* mRNAs progressively increased, while *ABCA1* mRNA progressively decreased in hypoxia, hypoxia/normoxia and intermittent hypoxia (Fig. 2b). The trend was peculiar of these three ABC transporters and not shared by other ABC family members (Additional file 1: Supplemental Fig. S2).

To explain the transcriptional changes of *ABCB1*, *ABCC1* and *ABCA1*, we investigated which transcription factors, commonly binding the promoters of these genes, followed the same variations of the three transporters in hypoxia, hypoxia/normoxia and intermittent hypoxia. Among the 38 transcription factors predicted to bind the promoters of *ABCB1*, *ABCC1* and *ABCA1* genes (Fig. 2c; Additional file 1: Supplemental Table S2), C/EBP-β emerged as the transcription factor most progressively increasing in hypoxia, hypoxia/normoxia and intermittent hypoxia (Fig. 2d), following the same trend of ABCB1 and ABCC1.

**Fig. 3:**
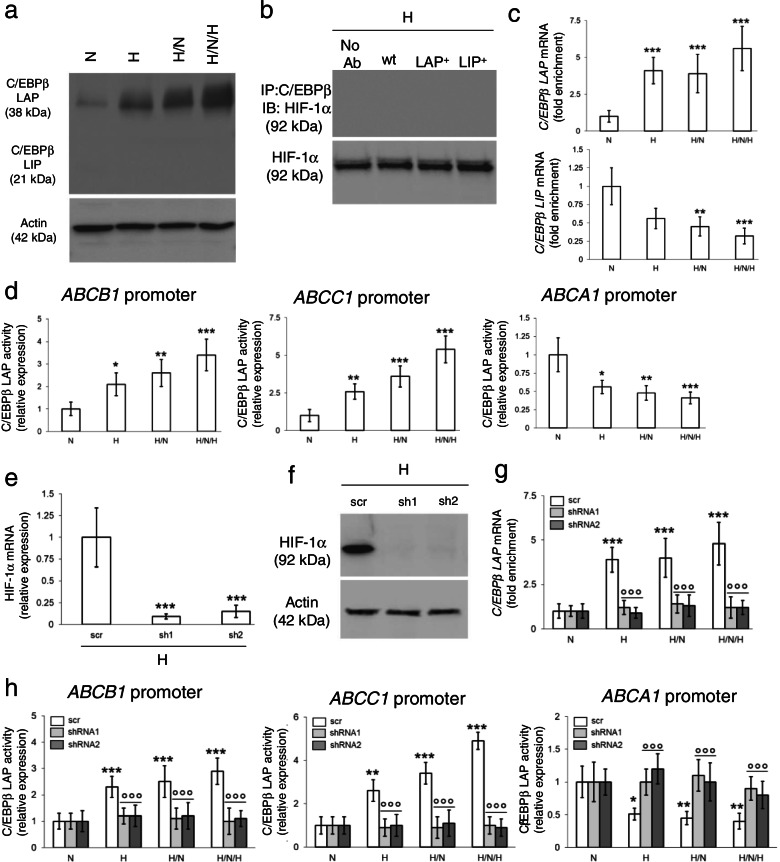
HIF-1α stabilizes C/EBP-β LAP mRNA, increasing ABCB1 and ABCC1, and decreasing ABCA1. NCI-H2228 cells were cultured as indicated in Fig. 1a. **a** Immunoblot of C/EBP-β LAP in whole cell extracts. Actin is included as control of equal protein loading. The image is representative of 1 out of 3 experiments. **b** Normoxic NCI-H2228 cells, wild-type (wt), overexpressing C/EBP-β LAP (LAP^+^) or overexpressing C/EBP-β LIP (LIP^+^), were lysed and immunoprecipitated with an anti-C/EBP-β antibody, recognizing both LAP and LIP isoforms, then immunoblotted for HIF-1α. An aliquot of the lysates before the immunoprecipitation was directly probed with the anti-HIF-1α antibody, to check that the protein was equally present. The image is representative of 1 out of 3 experiments. No Ab: wild-type cells subjected to immunoprecipitation without the anti-C/EBP-β antibody, as negative control. **c** RNA-IP with an anti-HIF-1α antibody, followed by RT-PCR amplification (in technical triplicates) with primers for C/EBP-β LAP (upper panel) or LIP (lower panel) isoforms. Data are means±SD (*n* = 4 biological replicates). **p < 0.01,***p < 0.001: H, H/N, H/N/H versus N cells. **d** ChIP of C/EBP-β on *ABCB1*, *ABCC1* and *ABCA1* promoter, in technical triplicates. Data are means±SD (*n* = 4 biological replicates). *p < 0.05,**p < 0.01,***p < 0.001: H, H/N, H/N/H versus N cells. **e-h** Hypoxic (H) NCI-H228 cells were treated with a non-targeting sequence (scr) or with two shRNAs (sh1, sh2) targeting HIF-1α. **e** HIF-1α mRNA level, measured by RT-PCR in technical triplicates. Data are means±SD (*n* = 4 biological replicates). ***p < 0.001: H, H/N, H/N/H versus N cells. **f** Immunoblot of HIF-1α. Actin is included as control of equal protein loading. The image is representative of 1 out of 3 experiments. **g** RNA-IP with an anti-HIF-1α antibody, followed by RT-PCR amplification (in technical triplicates) with primers for C/EBP-β LAP. Data are means±SD (*n* = 4 biological replicates). ***p < 0.001: H, H/N, H/N/H versus N cells; °°°*p* < 0.001: sh1/sh2-cells versus scr-cells. **h** ChIP of C/EBP-β on *ABCB1*, *ABCC1* and *ABCA1* promoter, in technical triplicates. Data are means±SD (*n* = 4 biological replicates). *p < 0.05,**p < 0.01,***p < 0.001: H, H/N, H/N/H versus N cells, °°°*p* < 0.001: sh1/sh2-cells versus scr-cells

Among the two splicing isoforms of C/EBP-β, LIP protein was undetectable, while LAP progressively increased from normoxia to more severe intermittent hypoxia (Fig. 3a). Co-immunoprecipitation experiments excluded that HIF-1α and C/EBP-β proteins interact in hypoxic cells (Fig. 3b), neither in wild-type cells nor in cells selectively overexpressing LAP or LIP (Additional file 1: Supplemental Fig. S3). Interestingly, both the C/EBP-β LAP (Additional file 1: Supplemental Fig. S4a) and LIP (Additional file 1: Supplemental Fig. S4b) mRNAs contained a hypoxia responsive element (HRE). RNA-IP assays indicated that, when immunoprecipitated, HIF-1α interacted with C/EBP-β mRNA: in the C/EBP-β mRNA bound by HIF-1α, we noticed a progressive increase in LAP mRNA and a progressive decrease in LIP mRNA in hypoxia, hypoxia/normoxia and intermittent hypoxia (Fig. 3c). Concurrently, the binding of LAP to the CAAT boxes of *ABCB1* and *ABCC1* promoters (Additional file 1: Supplemental Fig. S5a-b) increased (Fig. 3d), while the binding of LAP to the CAAT sequence of *ABCA1* promoter (Additional file 1: Supplemental Fig. S5c) decreased (Fig. 3d), suggesting that the transcriptional activity of LAP was opposite on *ABCB1/ABCC1* promoters and *ABCA1* promoter.

As proof of concept that HIF-1α increased LAP expression and activity in hypoxic cells, HIF-1α-silenced cells (Fig. 3e-f) did not enrich LAP mRNA (Fig. 3g) nor changed LAP transcriptional activity on *ABCB1*, *ABCC1* and *ABCA1* promoters (Fig. 3h) in normoxia, hypoxia, hypoxia/normoxia and intermittent hypoxia.

### C/EBP-β LAP determines chemo-immuno-resistance in intermittent hypoxia and is a negative prognostic factor in patients

**Fig. 4:**
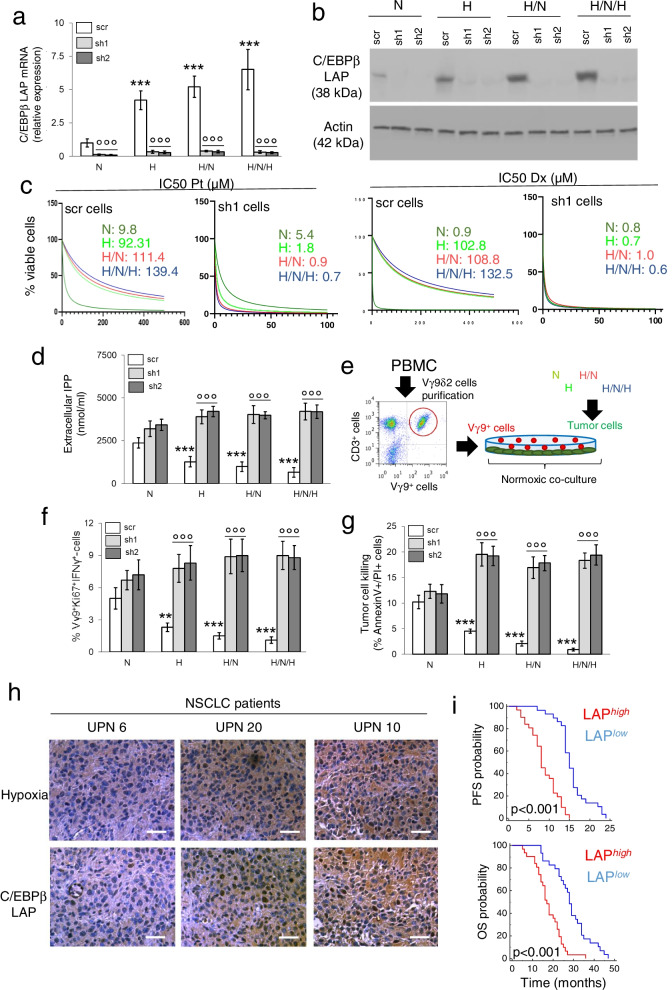
C/EBP-β LAP mediates chemo-immuno-resistance in hypoxic non-small cell lung cancer and is a negative prognostic factor. NCI-H2228 cells, cultured as indicated in Fig. 1a, were treated with a non-targeting sequence (scr) or with two shRNAs (sh1, sh2) targeting C/EBP-β. **a** C/EBP-β LAP mRNA level, measured by RT-PCR in technical triplicates. Data are means±SD (*n* = 4 biological replicates). ***p < 0.001: H, H/N, H/N/H versus N cells; °°°*p* < 0.001: sh1/sh2-cells versus scr-cells. **b** Immunoblot of C/EBP-β LAP. Actin is included as control of equal protein loading. The image is representative of 1 out of 3 experiments. **c** C/EBP-β-silenced cells (sh1) were treated for 48 h in normoxia (20% O_2_) with increasing concentrations (from 1 × 10^− 9^ to 1 × 10^− 5^ M) of cisplatin (Pt) and docetaxel (Dx). Cells treated with a non-targeting sequence (scr) were included as control. Cell viability was measured by a chemiluminescence-based assay, in technical quadruplicates (*n* = 3 biological replicates). Representative (inhibitor) vs. normalized dose-response curves and relative IC_50_, obtained with the GraphPad Prism 9 software. **d** Amount of released [^14^C]-IPP, considered an index of efflux, measured by liquid scintillation, in technical triplicates. Data are means±SD (*n* = 3 biological replicates). ***p < 0.001: H, H/N, H/N/H versus N cells; °°°*p* < 0.001: sh1/sh2-cells versus scr cells. **e.** Experimental scheme of the co-cultures of NCI-H2228 cells and Vγ9Vδ2 T cells. **f** Percentage of Ki67^+^IFN-γ^+^ Vγ9Vδ2 T cells collected after the co-cultures with the NCI-H2228 cells, evaluated by flow cytometry, in technical duplicates. Data are means±SD (*n* = 5 biological replicates). **p < 0.01,***p < 0.001: H, H/N, H/N/H versus N cells; °°°*p* < 0.001: sh1/sh2-cells versus scr-cells. **g** Percentage of annexin V^+^PI^+^ NCI-H2228 cells, as index of tumor cells killed by Vγ9Vδ2 T-cells, evaluated by flow cytometry, in technical duplicates. Data are means±SD (*n* = 5 biological replicates). ***p < 0.001: H, H/N, H/N/H versus N cells; °°°*p* < 0.001: sh1/sh2-cells versus scr-cells. **h** Representative immunohistochemistry images of intratumor hypoxic regions, measured with a pimonidazole-based probe, and C/EBP-β LAP-positive regions within 3 UPN NSCLC samples (63× objective, 20× ocular). Bar: 50 μm. **i** Patients were classified as LAP^low^ (*n* = 29) and LAP^high^ (*n* = 31) according to the median value of staining. PFS and OS probability were calculated using the Kaplan-Meier method. ***p < 0.001

In a complementary set of experiment, we silenced LAP, which was undetectable in normoxia, hypoxia, hypoxia/normoxia and intermittent hypoxia (Fig. 4a-b). In silenced cells, the IC_50_ of cisplatin and docetaxel was lower compared to parental cells (Fig. 4c; Additional file 1: Supplemental Fig. S6a-b), indicating that preventing the increase of LAP elicited by hypoxia reduces the chemoresistance mediated by ABCB1/ABCC1.

The decrease of ABCA1 in hypoxia, hypoxia/normoxia and intermittent hypoxia was associated to a progressive decrease in the efflux of IPP (Fig. 4d). Consistently, Vγ9Vδ2 T-cells, immuno-purified from PBMC and co-cultured in normoxic conditions with cancer cells, pre-conditioned by normoxia, hypoxia, hypoxia/normoxia and intermittent hypoxia (Fig. 4e), were progressively less proliferating and cytolytic, as indicated by the lower percentage of Ki67^+^IFNγ^+^ Vγ9Vδ2 T-cells (Fig. 4f) and by the lower amount of necro-apoptotic (i.e., annexin V^+^/PI^+^) cancer cells (Fig. 4g). C/EBP-β LAP was responsible for this immune-resistance because LAP silencing restored IPP efflux (Fig. 4d), Vγ9Vδ2 T-cells proliferation, activation (Fig. 4f) and immune-killing (Fig. 4g).

The molecular mechanism linking intermittent hypoxia to chemo-immuno-resistance was confirmed also in NCI-H1563 cells, which were chemo-immuno-sensitive in normoxia, but acquired a chemo-immuno-resistant phenotype comparable to NCI-H2228 cells in intermittent hypoxia (Fig. 1b-c). Indeed, also NCI-H1563 increased HIF-1α protein after continuous hypoxia, displayed a decrease after one hypoxic/normoxic shot and a further increase after intermittent hypoxia (Additional file 1: Fig. S7a). Classical HIF-1α-target genes – *EPO1*, *VEGFA* and *GLUT1* – followed HIF-1α protein expression, while *ABCB1* and *ABCC1* progressively increased, and *ABCA1* progressively decreased (Additional file 1: Fig. S7b). Continuous hypoxia, one hypoxic/normoxic shot and intermittent hypoxia progressively stabilized C/EBP-β LAP mRNA (Additional file 1: Fig. S7c) and protein (Additional file 1: Fig. S7d), while they progressively decreased C/EBP-β LIP mRNA, according to RNA-IP assays (Additional file 1: Fig. S7c). In line with these results, LAP binding to *ABCB1* and *ABCC1* promoters progressively increased, LAP binding to *ABCA1* promoter progressively decreased (Additional file 1: Fig. S7e), explaining the up-regulation of *ABCB1* and *ABCC1* mRNA, and the downregulation of the *ABCA1* mRNA (Additional file 1: Fig. S7b). This mechanism also explained the increase in IC_50_ of cisplatin and docetaxel in NCI-H1563 cells exposed to hypoxia, hypoxia/normoxia and intermittent hypoxia (Additional file 1: Fig. S7f) that resulted in chemoresistance. In parallel, the IPP efflux was reduced (Additional file 1: Fig. S7g), as well as the proliferation and expansion of Vγ9Vδ2 T-lymphocytes (Additional file 1: Fig. S7h) and their immune-killing against NCI-H1536 cells (Additional file 1: Fig. S7i).

Moreover, to validate the clinical meaning of C/EBP-β LAP, we analyzed a cohort of 60 prospectively recruited NSCLC patients at stage III/IV, candidate to receive adjuvant chemotherapy with cisplatin/carboplatin as first-line treatment (Additional file 1: Supplemental Table S3). C/EBP-β LAP was detected in all patient biopsies, with different intensity (Additional file 1: Supplemental Table S3), and was higher in the most hypoxic tumors (Fig. 4h). The median of LAP staining intensity was used to dichotomize patients in LAP^*low*^ and LAP^*high*^ groups. Notably, the LAP^*high*^ group had significantly lower PFS and OS (Fig. 4i).

### Mitochondrial ROS promote C/EBP-β LAP stabilization during intermittent hypoxia

During intermittent hypoxia, mitochondria are exposed to periods of O_2_ abundance and O_2_ shortage. The first situation determines a regular electron flux though the ETC, culminating in the reduction of O_2_ into H_2_O and in the production of ATP via OXPHOS. During the phase of O_2_ deprivation, NADH and FADH_2_ cofactors accumulated in the reduced form, the electron flux and H^+^ gradient are impaired, and the synthesis of ATP is reduced. A new cycle of re-oxygenation pushes the oxidation of NADH and FADH_2_, as well as the electron flux through ETC. This process is often paralleled by uncomplete reduction of O_2_ and generation of partially reduced O_2_ species that produce mtROS, damaging mitochondria and further impairing the efficiency of ETC coupled with OXPHOS. This triggers a vicious circle enhancing the generation of mtROS [4]. Since mtROS mediate part of HIF-1α physiological effects [30], we investigated if they were also involved in LAP stabilization.

**Fig. 5:**
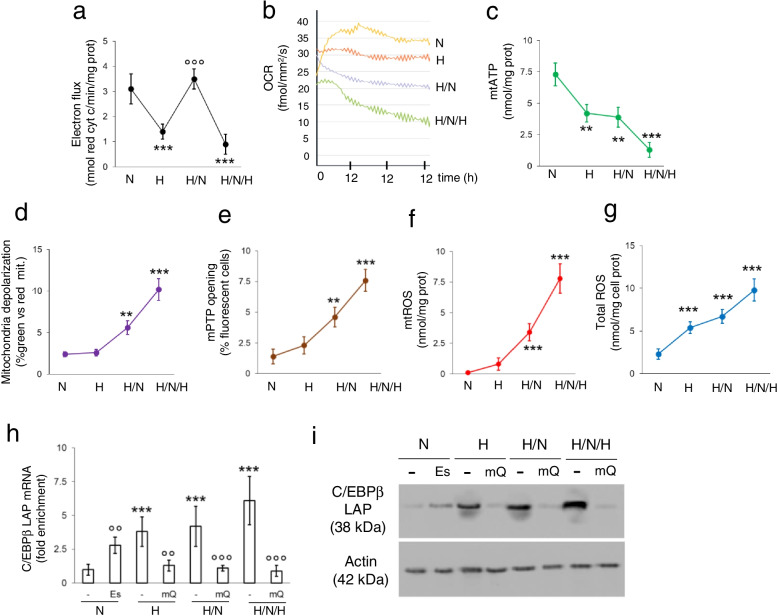
Mitochondrial ROS stabilize C/EBP-β LAP in hypoxic non-small cell lung cancer. NCI-H2228 cells were cultured as indicated in Fig. 1a. When indicated, 50 μM elesclomol (Es) or 0.4 μM mitoquinol (mQ) were added to the normoxic or hypoxic cultures. **a-g** Mitochondrial electron flux (**a**), O_2_ consumption (**b**), ATP (**c**), depolarization (**d**), mPTP opening (**e**), mtROS (**f**) and total ROS (**g**), measured in technical triplicates. Data are means±SD (*n* = 4 biological replicates). **p < 0.001,***p < 0.001: H, H/N/H versus N cells; °°°*p* < 0.001: H/N cells versus H cells. **h** RNA-IP with an anti-HIF-1α antibody, followed by RT-PCR amplification (in technical triplicates) with primers for C/EBP-β LAP (upper panel) or LIP (lower panel) isoforms. Data are means±SD (*n* = 4 biological replicates). ***p < 0.001: H, H/N, H/N/H versus N cells; °°*p* < 0.01,°°°*p* < 0.001: Es−/mQ-treated cells versus untreated cells. **i** Immunoblot of C/EBP-β LAP in whole cell extracts. Actin is a control of equal protein loading. The image is representative of 1 out of 3 experiments

As expected, the ETC flux dropped after 24 h of hypoxia, was rescued by 12 h of normoxia following 12 h of hypoxia, but it was dramatically low after intermittent hypoxia (Fig. 5a). The real-time O_2_ consumption in live cells showed a progressively decrease in cells exposed to continuous normoxia, one shot of hypoxia followed by normoxia, intermittent hypoxia (Fig. 5b), indicating that repeated hypoxic cycles irreversibly compromised the efficiency of OXPHOS. Accordingly, mitochondrial ATP levels irreversibly decreased (Fig. 5c). Also, mitochondria lost their membrane potential as indicated by the higher percentage of green mitochondria after JC-1 staining (Fig. 5d) and by the opening of mPTP (Fig. 5e), an event common in mitochondria metabolically damaged after ischemia/reperfusion and caused by increased mitochondrial oxidative stress [31]. Indeed, we detected an increase of mtROS (Fig. 5f), paralleled by an increase in total ROS (Fig. 5g), indicating a diffusion of ROS from the mitochondria to the cytosol and/or the generation of radical species within the cytosol.

As far as the antioxidant defenses were concerned, moving from normoxia to continuous hypoxia, one hypoxia/normoxia shot and intermittent hypoxia we detected a significant increase in the activity of mitochondrial SOD2 (Additional file 1: Supplemental Fig. S8a), while the cytosolic SOD1 (Additional file 1: Supplemental Fig. S8b) and catalase (Additional file 1: Supplemental Fig. S8c) increased at lesser extent. The ratio between GHS/GSS was progressively decreased (Additional file 1: Supplemental Fig. S8d), while the activity of GPX was progressively increased (Additional file 1: Supplemental Fig. S8e). However, cell viability did not significantly decrease in continuous hypoxia, one hypoxia/normoxia shot and intermittent hypoxia (Additional file 1: Supplemental Fig. S8f).

To assess the functional effect of mtROS on C/EBP-β LAP stabilization, we treated normoxic NCI-H2228 cells with the ETC-targeting and pro-oxidant agent elesclomol [32], at a concentration that increased mtROS to the same level of intermittent hypoxia (Additional file 1: Supplemental Fig. S9a), and hypoxic cells with the mtROS scavenger mitoquinol (mitoQ) [33], to reduce ROS at the same levels of normoxic cells (Additional file 1: Supplemental Fig. S9b). Notably, mitoQ prevented the stabilization of LAP mRNA mediated by HIF-1α (Fig. 5h) and reduced LAP protein (Fig. 5i) in hypoxia, hypoxia/normoxia and intermittent hypoxia.

**Fig. 6:**
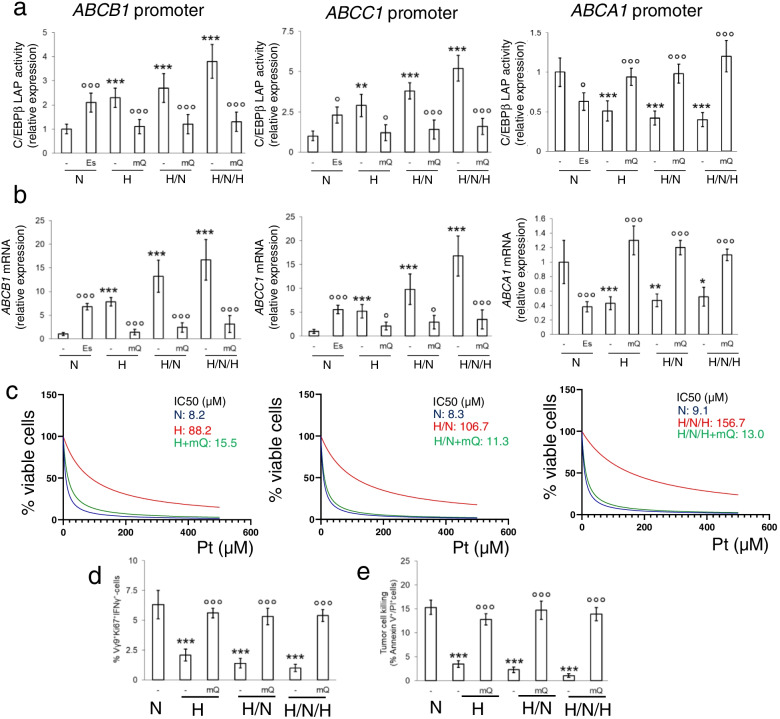
Mitochondrial ROS scavenging prevents LAP-induced up-regulation of ABC transporters and chemo-immuno-resistance. NCI-H2228 cells were cultured as indicated in Fig. 1a. When indicated, 50 μM elesclomol (Es) or 0.4 μM mitoquinol (mQ) were added to the normoxic or hypoxic cultures. **a-b** ChIP of C/EBP-β on *ABCB1*, *ABCC1* and *ABCA1* promoter (**a**) and *ABCB1*, *ABCC1*, *ABCA1* mRNAs (**b**), measured by RT-PCR, in technical triplicates. Data are means±SD (*n* = 4 biological replicates). *p < 0.05,**p < 0.01,***p < 0.001: H, H/N, H/N/H versus N cells; °*p* < 0.05 °°°*p* < 0.001: Es/mQ-treated cells versus untreated cells. **c** After normoxic and hypoxic cultures, cells were treated for 48 h in normoxia (20% O_2_) with increasing concentrations (from 1 × 10^− 9^ to 1 × 10^− 5^ M) of cisplatin (Pt). Cell viability was measured in technical quadruplicates (*n* = 3 biological replicates). Representative (inhibitor) vs. normalized dose-response curves and relative IC_50_, obtained with the GraphPad Prism 9 software. **d-e** Percentage of Ki67^+^IFN-γ^+^ Vγ9Vδ2 T cells (**d**) and percentage of annexin V^+^PI^+^ NCI-H2228 cells (**e**), as index of tumor cells killed by Vγ9Vδ2 T-cells, collected after the co-cultures with the NCI-H2228 cells, evaluated by flow cytometry, in technical duplicates. Data are means±SD (*n* = 5 biological replicates). ***p < 0.001: H, H/N, H/N/H versus N cells; °°°*p* < 0.001: mQ-treated cells versus untreated cells

MitoQ abrogated the hypoxia-induced transcriptional activity of LAP (Fig. 6a), reduced *ABCB1* and *ABCC1* mRNAs, and increased *ABCA1* mRNA (Fig. 6b) to levels comparable to normoxia. By contrast, normoxic cells treated with elesclomol displayed the same amount of LAP, activity and expression of ABC transporters of hypoxic cells (Fig. 6a-b). Consistently, mitoQ reduced the IC_50_ of cisplatin and docetaxel in cells subjected to hypoxia, hypoxia/normoxia or intermittent hypoxia (Fig. 6c; Additional file 1: Supplemental Fig. S10), maintaining high the amount of active and proliferating Vγ9Vδ2 T-cells (Fig. 6d), and the tumor killing (Fig. 6e) in all the hypoxic conditions. Again, also in chemo-immuno-sensitive NCI-H1563 cells, the culture in hypoxia, hypoxia/normoxia or intermittent hypoxia produced a progressive increase in mtROS (Additional file 1: Supplemental Fig. S11a), effectively scavenged by mitoQ (Additional file 1: Supplemental Fig. S11b). MitoQ reduced the IC_50_ of cisplatin and docetaxel (Additional file 1: Supplemental Fig. S11c), increased the activation and immune-killing of Vγ9Vδ2 T-lymphocytes to levels comparable to hypoxic conditions (Additional file 1: Supplemental Fig. S11d-e), reproducing the same effects observed in constitutively chemo-immuno-resistant NCI-H2228 cells.

### Scavenging mitochondrial ROS induces chemo-immuno-sensitization in hypoxic immuno-xenografts

**Fig. 7:**
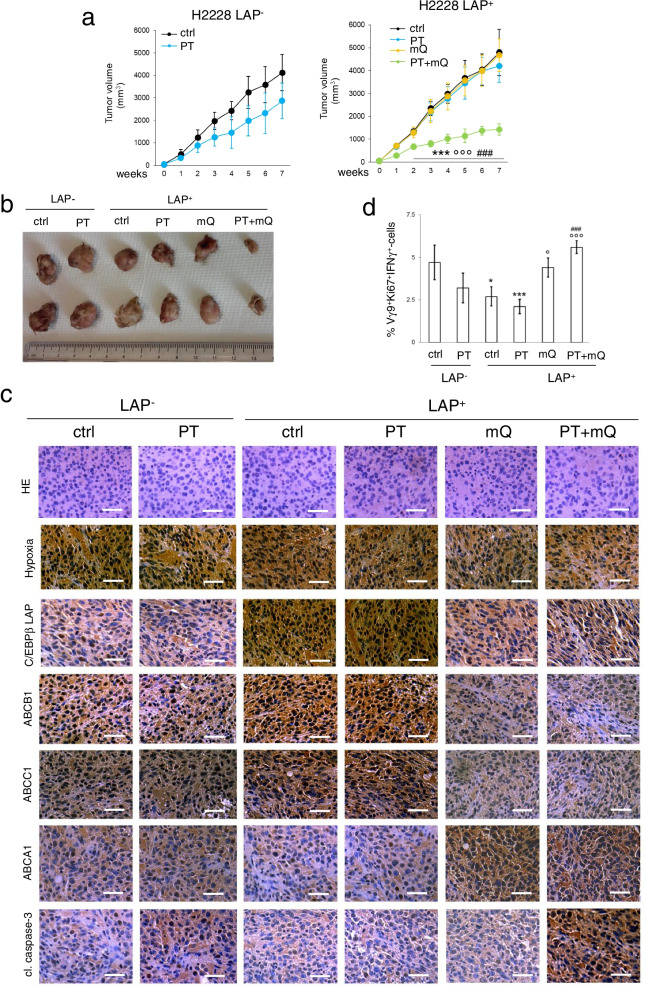
Scavenging mitochondrial ROS rescues chemo-immune-resistance in immuno-xenografts. 1 × 10^6^ C/EBP-β LAP-overexpressing cells were injected subcutaneously in Hu-CD34^+^NSG mice. When tumor reached the volume of 50 mm^3^, animals (*n* = 6/group) were randomized and treated for 6 weeks as it follows: 1) vehicle (ctrl) group, treated with 0.1 ml saline solution intravenously (i.v.), once a week; 2) cisplatin (PT) group, treated with 2 mg/kg cisplatin i.v., once a week; 3) mitoquinol (mQ) group, treated with 100 mg/kg daily via oral gavage; 4) cisplatin + mitoquinol (PT + mQ) group, treated with 2 mg/kg cisplatin i.v., once a week. and 100 mg/kg mitoquinol daily via oral gavage. To induce LAP intratumourally, 1 mg/ml doxycycline was added daily to the drinking water (LAP^+^ mice). **a** Tumor growth was monitored by caliper. ***p < 0.001: LAP^+^PT + mQ-group vs. LAP^−^ctrl-group; °°°*p* < 0.001: LAP^+^ T + mQ-group vs LAP^+^ctrl-group; ^###^*p* < 0.001: LAP^+^PT + mQ-group vs LAP^+^PT-group (weeks 2–7). **b** Representative photographs of tumors from each group of treatment. **c** Representative immunohistochemical microphotographs of hematoxylin/eosin (HE) stained sections, sections immunostained for the pimonidazole hypoxic-probe or for the indicated proteins (63× objective, 20× ocular). Bar: 50 μm. **d** Percentage of intratumor Vγ9δ2 T-lymphocytes among all CD3^+^T-lymphocyte-infiltrating cells, measured by flow cytometry. Data are means±SD (*n* = 6 tumors). *p < 0.05,***p < 0.001: LAP^+^PT + mQ-group vs. LAP^−^ctrl-group; °*p* < 0.05,°°°*p* < 0.001: LAP^+^mQ/PT + mQ-group vs LAP^+^ctrl-group; ^###^*p* < 0.001: LAP^+^PT + mQ-group vs LAP^+^PT-group

The efficacy of mitoQ was validated in Hu-CD34^+^NSG, bearing a reconstituted human immune system including Vγ9Vδ2 T-cells in peripheral blood [34], and NCI-H2228 tumors, engineered with a doxycycline-inducible LAP expression vector [13]. Preliminary dose-dependent experiments identified the dosage of 100 mg/kg mQ as the lowest dose that significantly reduced the growth of LAP-overexpressing tumors in combination with cisplatin, compared with cisplatin alone (Additional file 1: Supplemental Fig. S12). This dosage was chosen for the following experiments. In the absence of LAP induction cisplatin only slightly reduced tumor growth (Fig. 7a-b; Additional file 1: Supplemental Fig. S13a), in agreement with the chemoresistant phenotype of this cell line (Fig. 1b). The induction of LAP made tumors more resistant to cisplatin. MitoQ, which did not exert anti-tumor effects, dramatically reduced tumor growth in combination with cisplatin (Fig. 7a-b; Additional file 1: Supplementary Fig. S13a). NCI-H2228 tumors, which were hypoxic (Fig. 7c), had detectable levels of ABCB1 and ABCC1, and very low levels of ABCA1, not modified by cisplatin (Fig. 7c; Additional file 1: Supplemental Fig. 13b). Intratumor LAP induction increased ABCB1 and ABCC1, and decreased ABCA1. Notably, mitoQ reduced LAP, ABCB1 and ABCC1, and increased ABCA1, both alone and with cisplatin (Fig. 7c; Additional file 1: Supplemental Fig. 13b). Cisplatin moderately activated the caspase 3 in NCI-H2228 tumors; this effect was abrogated in LAP-overexpressing tumors and restored by mitoQ (Fig. 7c; Additional file 1: Supplemental Fig. 13b). In line with the modulation of ABCA1 levels, intratumor activated Ki67^+^IFNγ^+^ Vγ9Vδ2 T-cells were lower upon LAP induction, but their percentage was increased by mitoQ (Fig. 7d).

Furthermore, neither alone nor in combination with cisplatin, mitoQ was toxic, according to animal weight (Additional file 1: Supplemental Fig. 13c), *post-mortem* analysis of liver, kidney and spleen (Additional file 1: Supplemental Fig. 13d), and hematochemical parameters assessing bone marrow, liver and muscle/heart toxicities (Additional file 1: Supplemental Table S4). At the end of the treatment cisplatin increased creatinine, but the presence of mitoQ reduced such increase (Additional file 1: Supplemental Table S4).

## Discussion

The growth in stably hypoxic conditions determines chemoresistance [4] and impairs the activity of effector T-lymphocytes [35], but less is known about the effects of intermittent hypoxia. Two orders of intermittent hypoxia have been reported in tumors: a rapidly intermittent hypoxia, occurring 2–5 times/hour, and a prolonged intermittent hypoxia, with cycles of hours, caused by vascular reshaping [2]. In animal models of NSCLC this chronic intermittent hypoxia induces aggressive tumors, characterized by the activation of HIF-1α transcriptional program, apoptosis inhibition, stemness, epithelial mesenchymal transition and drug resistance [36, 37].

With the goal of identifying molecular circuitries determining resistance to chemotherapy and immune-killing with the increase of hypoxia severity, we set up models of progressively more severe intermittent hypoxia [2]. After a high-throughput screening of NSCLC cells, we found that intermittent hypoxia progressively increased the drug efflux transporters ABCB1 and ABCC1, and decreased the immuno-sensitizer transporter ABCA1, in a cell line-independent way. We first hypothesized that the transcriptional changes of ABC transporters were mediated by HIF-1α that was increased after intermittent hypoxia more than after one shot of continuous hypoxia, but reduced after one hypoxia/normoxia cycle, likely because of the activity of PHD2 enzyme, which is active during re-oxygenation [38]. Intriguingly, while the expression of HIF-1α direct target genes (*EPO1*, *VEGFA*, *GLUT1*) followed HIF-1α levels, the levels of *ABCB1*, *ABCC1* and *ABCA1* mRNAs did not vary as HIF-1α protein. We thus hypothesized that other transcription factors, activated by HIF-1α or hypoxia-related pathways, mediated these transcriptional changes in ABC transporters. Indeed, in blood-brain barrier exposed to chronic intermittent hypoxia, ABCB1 was up-regulated by both HIF-1α and Nrf-2, which is stabilized by the ROS generated in hypoxia [39]. Among the 38 transcription factors commonly binding the promoters of *ABCB1, ABCC1* and *ABCA1*, C/EBP-β emerged as the factor most progressively increasing in chronic hypoxia, hypoxia/normoxia and intermittent hypoxia. In agreement with our findings, in human endothelial cells intermittent hypoxia increases C/EBP-β, which cooperates with HIF-1α to activate its transcriptional program [40]. Instead of a protein-protein interaction between HIF-1α and C/EBP-β, we found that in NSCLC cells HIF-1α protein was progressively more associated to C/EBP-β mRNA in hypoxia, hypoxia/normoxia and intermittent hypoxia, favoring the production of LAP splicing isoform. This data was consistent with the high levels of HIF-1α in cells growing in continuous hypoxia and intermittent hypoxia. It was less expected after one hypoxic/normoxic cycle, when cells had lower HIF-1α, but high LAP mRNA. It is likely that the residual levels of HIF-1α after the first hypoxic cycle were sufficient to stabilize LAP mRNA. Indeed, the knock-out of HIF-1α proved that the downstream biological effects mediated by C/EBP-β LAP were HIF-1α-dependent in all the hypoxic conditions tested. Although HIF-1α is known as a transcriptional regulator more than a splicing controller, one recent work reported that HIF-1α regulates the alternative splicing of SLC35A3 mRNA in pancreatic adenocarcinoma [41], paving the way to consider HIF-1α also as a post-transcriptional regulator, as it occurs in the case of C/EBP-β splicing. Interestingly, two HREs are present in the C/EBP-β mRNA: the mRNA encoding for LAP isoform contains both HREs, the mRNA encoding for LIP contains only the second one. Since the production of LAP and LIP occurs by alternative splicing [13], we hypothesize that HIF-1α binding to these HREs affects the recruitment and activity of specific proteins of splicing machinery, and/or of proteins involved in the stability of C/EBP-β mRNA. Our experimental data suggested that the binding of HIF-1α to the HRE in the 5′ portion (contained in the mRNA sequence encoding for LAP, but not for LIP), reduced the splicing activity generating LIP and/or stabilized the C/EBP-β mRNA, leading to the translation of the full-length isoform LAP. The binding of HIF-1α to the HRE in the 3′ portion (contained in the mRNA sequence encoding for LIP) reduced the splicing activity generating LIP as well, and/or destabilizes the 3′ portion of C/EBP-β mRNA, encoding for LIP. The result is an increased LAP/LIP ratio at mRNA and proteins levels, particularly pronounced when HIF-1α accumulates, as in case of intermittent hypoxia. This change in LAP/LIP ratio may explain the progressive up-regulation of ABCB1 [13] and ABCC1 [9], and the consequent multidrug resistant phenotype.

In addition, the activation of HIF-1α/LAP axis down-regulated ABCA1, causing lower killing by Vγ2Vδ9 T-cells. Hypoxia impairs the effector functions of γδ T-lymphocytes against glioblastoma cells [42], in line with the general immuno-suppression observed in the hypoxic tumor environment [35, 43]. The novelty of our results, however, rely on the demonstration that the hypoxic pre-conditioning of tumor cells prevents the expansion and activation of Vγ2Vδ9 T-cells grown in normoxic conditions, suggesting that cancer cells maintain a “hypoxic memory”. This memory was due to the intratumor activation of HIF-1α/LAP axis, which reduced the expression of ABCA1 and the efflux of IPP.

The hypoxia-mediated induction of LAP had a strong clinical meaning: indeed, in NSCLC patients candidate to receive cisplatin/carboplatin as first-line therapy, LAP expression correlated well with higher intratumour hypoxia, lower PFS, indicative of poor response to chemotherapy, and lower OS, indicative of bad prognosis. Notably, a recent work demonstrated that in triple negative breast cancer hypoxia selected ROS-resistant clones more prone to produce lung metastases, and induced a hypoxic gene reprogramming associated with a lower survival [44]. Since metastatic triple negative breast cancer is typically chemoresistant [45], we believe that a similar situation may occur in hypoxic NSCLC cells, where intracellular ROS favor the maintenance of chemoresistant clones.

We thus focused on the role of ROS, which are increased in intermittent hypoxia [46], as potential inducers of the chemo-immuno-resistance mediated by HIF-1α/LAP axis. The mitochondrial parameters analyzed indicated that a peculiarity of intermittent hypoxia is the progressively decrease in O_2_ consumption and ATP synthesis, despite a temporary increase in ETC during the phases of re-oxygenation. This drop in ATP and O_2_ consumption, paralleled by an intermittently functioning ETC [4], unequivocally produces O_2_ radicals that damage mitochondria [31]. Indeed, in line with endothelial cells [47] and neurons [48], also in NSCLC cells intermittent hypoxia impaired OXPHOS, increased mtROS and mitochondrial damage. Interestingly, the analysis of the anti-oxidant defenses of NCI-H2228 cells suggested that the increase in mtROS caused by the impaired ETC produced a compensatory activation of SOD2, which is localized near the primary source of ROS. The increase in cytosolic ROS also produced a compensatory increase of cytosolic anti-oxidant enzymes, as SOD1 and catalase. Similarly, intracellular ROS oxidized GSH via the GPX. The increase in mtROS and cytosolic ROS was not apparently cytotoxic for NCI-H2228 cells, as demonstrated by the absence of significant changes in cell viability, suggesting that an effective compensation of the oxidative stress generated by the impaired ETC. Noteworthy, non-harmful levels of mtROS have been indicated as mediators of chemoresistance [49], since they train cells to become more resilient to stressing conditions, including chemotherapeutic drugs [50]. We cannot exclude that this oxido-reductive phenotype contributed to generate the resistance to cisplatin observed in intermittent hypoxia. This hypothesis was supported by the chemosensitizing effect achieved by the mtROS scavenger mitoQ. Indeed, the use of mitoQ and of its antagonist elesclomol, a mtROS generator, proved that mtROS stabilized C/EBP-β LAP mRNA, modulating the expression of ABCB1, ABCC1 and ABCA1, the resistance to chemotherapy and immune-killing.

Our experimental assays were phenocopied in two different NSCLC cell lines: the NCI-H2228 cells, chosen as a prototype of constitutively chemo-immuno-resistant cells in normoxia whose resistant phenotype is further worsened by intermittent hypoxia, and the NCI-H1563 cells, chosen because they display a chemo-immuno-sensitive phenotype in normoxic conditions, but they acquire a chemo-immuno-resistant phenotype comparable to NCI-H2228 in intermittent hypoxia. Our results demonstrated that the mechanisms linking intermittent hypoxia and chemo-immuno-resistance (i.e. the activation of mtROS/ HIF-1α C/EBP-β LAP axis) were the same in NSCLC cells, either sensitive or resistant under normoxic conditions, and that the chemo-immuno-sensitizer strategies proposed were effective in both cases.

Finally, the chemo-immuno-resistant phenotype induced by the mtROS/LAP axis and the efficacy of mitoQ as chemo-immuno-sensitizer was recapitulated in humanized mice, bearing NCI-H2228 tumors with a diffuse intratumor hypoxia and an inducible C/EBP-β LAP expression system. MitoQ alone did not exert any antitumor effects, in line with previous findings on triple negative breast cancer [51]. However, we identified a combination treatment of cisplatin and mitoQ that rescued the antitumor efficacy of cisplatin and restored the intratumor activity of Vγ2Vδ9 T-cells also against chemo-immuno-resistant LAP-overexpressing NCI-H2228 tumors. This observation is in partial agreement with the findings of Capeloa et al., who demonstrated that mitoQ, combined with chemotherapy, reduced the number of metastases [51]. We hypothesize that the combination of mitoQ *plus* chemotherapy eradicated those cells resistant to chemotherapy that may produce recurrence and metastases, in triple negative breast cancer, as well as in cisplatin-resistant NSCLC. Notably, the combination therapy was not more toxic than cisplatin alone: mitoQ even limited the increase in creatinine induced by cisplatin, leading to hypothesize a potential protection toward cisplatin-induced nephrotoxicity. These data suggest a good therapeutic window for the combination of cisplatin *plus* mitoQ, noteworthy of further investigations at clinical level.

## Conclusions

**Fig. 8:**
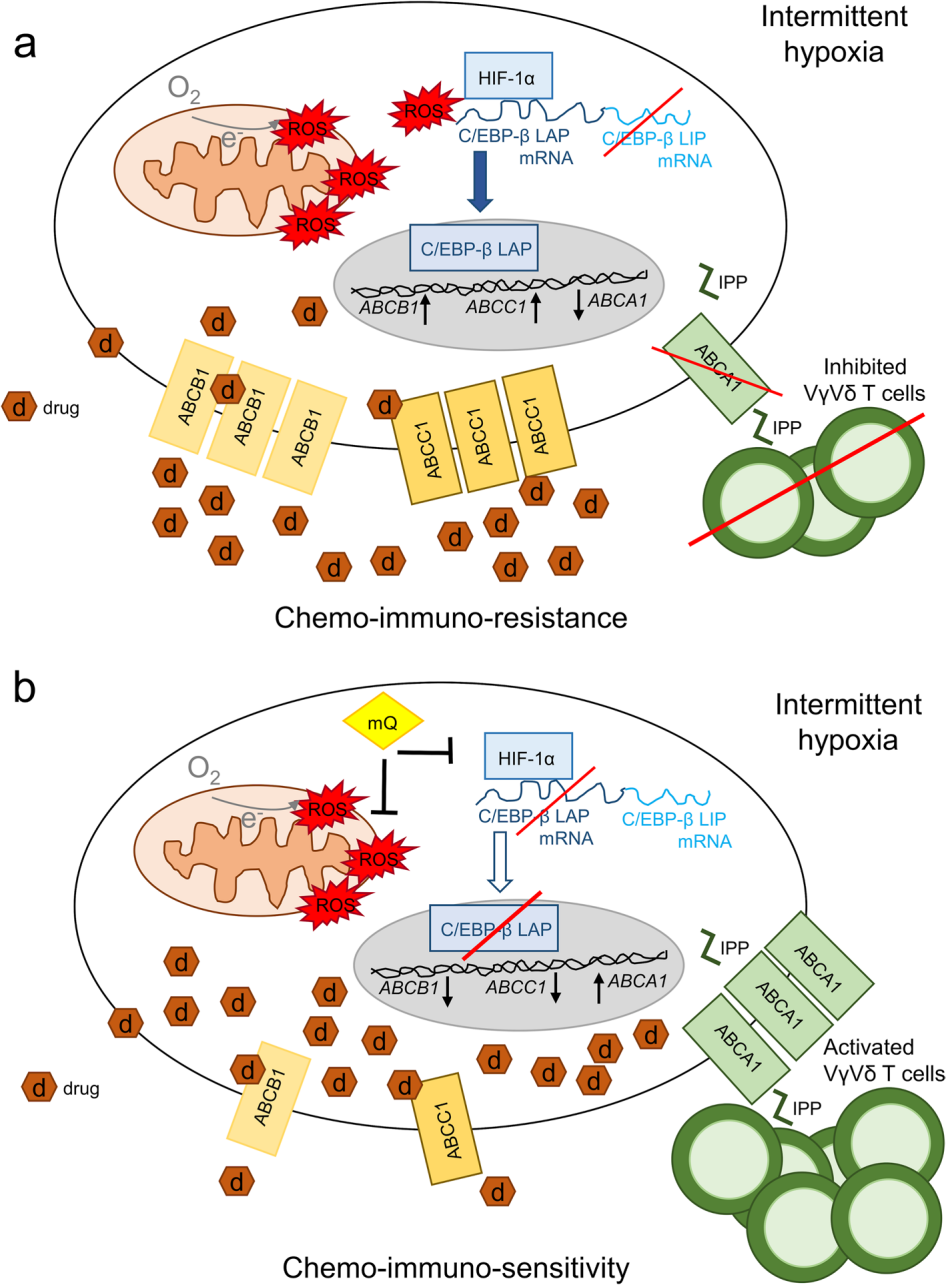
Mitochondrial ROS induce chemo-immune-resistance in hypoxic tumors by up-regulating the HIF-1α/C/EBP-β axis. **a** Intermittent hypoxia, often experimented by solid tumors, reduces the efficiency of mitochondrial electron transport chain, increases mitochondrial depolarization and damage, leading to the high production of reactive oxygen species (ROS). Mitochondrial ROS stabilize the interaction of the hypoxia-inducible factor-1α (HIF-1α) with the mRNA of the transcription factor CCAAT enhancer binding protein beta (C/EBP-β). This interaction increases C/EBP-β LAP and decreases C/EBP-β LIP isoform. LAP up-regulates *ABCB*1 and *ABCC1*, and decrease *ABCA1* at transcriptional level. The high levels of ABCB1 and ABCC1 induce the efflux of chemotherapeutic drugs (d), causing chemoresistance. The low level of ABCA1 prevents the efflux of isopentenyl pyrophosphate (IPP) and the activation of the anti-tumor Vγ9Vδ2 T-cells, determining immuno-resistance. **b** Scavenging mitochondrial ROS with mitoquinol (mitoQ) disrupts the HIF-1α C/EBP-β axis, increases the LIP/LAP ratio, decreasing the drug efflux transporters ABCB1 and ABCC1, and increasing the immuno-sensitizer transporter ABCA1. As a result, hypoxic cells were more sensitive to chemotherapy and to Vγ9Vδ2 T-cell immune-killing

We unveiled a cancer cell autonomous mechanism that induces the simultaneous resistance to chemotherapy and immune-killing in hypoxic NSCLCs. By impairing the OXPHOS and increasing mtROS, intermittent hypoxia stabilizes the interaction between HIF-1α and C/EBP-β mRNA, favoring the generation of C/EBP-β LAP isoform that up-regulates ABCB1 and ABCC1, causing chemoresistance, and down-regulates ABCA1, promoting a low anti-tumor activity of Vγ9Vδ2 T-cells. Preventing this cascade with mtROS scavengers as mitoQ induces chemo-immuno-sensitization of hypoxic tumors (Fig. 8). In a translational perspective, our work has at least two implications. First, C/EBP-β LAP, which is well detected by immunohistochemical analyses, could be included in the diagnostic workflow of NSCLC, as a new predictive and prognostic factor for patients candidate to receive chemotherapy. Second, we propose to repurpose mitochondrial ROS scavengers as mitoQ, currently under evaluation in inflammatory-degenerative neurological, cardiovascular, liver and kidney diseases, characterized by ischemia and re-oxygenation [52], as chemo immuno-sensitizer agents. Improving chemotherapy efficacy means increasing the efficacy of one of the main therapeutic options for advanced NSCLC. At the same time, since γδ T-cells are the most favorable prognostic tumor-infiltrating population in lung cancer [21], their activation in hypoxic tumors, where other treatments as immune checkpoint inhibitors or CAR T-cells have limited efficacy, may represent an innovative immunotherapeutic approach.

## Supplementary Information

**Additional file 1: Table S1.** RT-PCR primers list. **Table S2.** Transcription factors binding ABCB1, ABCC1 and ABCA1 promoters. **Table S3.** Patient clinical-pathological features. **Fig. S1.** Intermittent hypoxia increases the IC50 of cisplatin and docetaxel in non-small cell lung cancer NCI-H2228 cells. NCI-H2228 cells were cultured in the following conditions: normoxia (at 20% O2 for 24 h, N), hypoxia (at 1% O2 for 24 h, H), hypoxia/normoxia (12 h at 1% O2 followed by 12 h at 20% O2, H/N), hypoxia/normoxia/hypoxia or intermittent hypoxia (12 h at 1% O2 followed by 12 h at 20% O2 and 12 h at 1% O2, H/N/H), then incubated for 48 h in normoxia (20% O2) with increasing concentrations (from 1 × 10− 9 to 1 × 10− 5 M) of cisplatin (Pt, a) and docetaxel (Dx, b). Cell viability was measured by a chemiluminescence-based assay, in technical quadruplicates (*n* = 4 biological replicates). Representative [inhibitor] vs. normalized dose-response curves and relative IC50, obtained with the GraphPad Prism 9 software. **Fig. S2.** Effects of intermittent hypoxia on ABC transporters expression. NCI-H2228 cells were cultured in the following conditions: normoxia (at 20% O2 for 24 h, N), hypoxia (at 1% O2 for 24 h, H), hypoxia/normoxia (12 h at 1% O2 followed by 12 h at 20% O2, H/N), hypoxia/normoxia/hypoxia or intermittent hypoxia (12 h at 1% O2 followed by 12 h at 20% O2 and 12 h at 1% O2, H/N/H). The expression level of the indicated ABC transporters was measured by a RT-PCR array and is represented in a colorimetric scale. The expression of each ABC transporter in normoxic cells was considered 1; the relative expression in the other experimental conditions is indicated in the heatmap. **Supplemental Fig. S3.** Overexpression of C/EBP-β LAP and LIP. Normoxic NCI-H2228 cells, wild-type (wt), overexpressing C/EBP-β LAP (LAP+) or overexpressing C/EBP-β LIP (LIP+) were lysed and immunoblotted with an anti-C/EBP-β antibody, recognizing both LAP and LIP isoforms. Actin is included as control of equal protein loading. The image is representative of 1 out of 3 experiments. **Supplemental Fig. S4.** C/EBP-β mRNA sequence. Sequence of C/EBP-β LAP (a) and LIP (b) mRNA. The hypoxia-response element (HRE) is highlighted in cyano, the ATG codon of LIP mRNA in green. **Supplemental Fig. S5.** ABCB1, ABCC1 and ABCA1 promoter sequences. Sequences of ABCB1 (a), ABCC1 (b) and ABCA1 (a) promoters. The putative binding sites for C/EBP-β (CAAT boxes) are highlighted in yellow. **Supplemental Fig. S6.** Effects of C/EBP-β silencing on sensitivity to cisplatin and docetaxel. NCI-H2228 cells, stably silenced with the C/EBP-β-targeting sequence #2 (sh2), were cultured in the following conditions: normoxia (at 20% O2 for 24 h, N), hypoxia (at 1% O2 for 24 h, H), hypoxia/normoxia (12 h at 1% O2 followed by 12 h at 20% O2, H/N), hypoxia/normoxia/hypoxia or intermittent hypoxia (12 h at 1% O2 followed by 12 h at 20% O2 and 12 h at 1% O2, H/N/H). After normoxic and hypoxic cultures, cells were treated for 48 h in normoxia (20% O2) with increasing concentrations (from 1 × 10− 9 to 1 × 10− 5 M) of cisplatin (Pt, a) and docetaxel (Dx, b). Cell viability was measured by a chemiluminescence-based assay, in technical quadruplicates (*n* = 3 biological replicates). Representative [inhibitor] vs. normalized dose-response curves and relative IC50, obtained with the GraphPad Prism 9 software. The dose-response curves and relative IC50 in NCI-H2228 cells treated with a non-targeting sequence (scr) shRNA sequence are reported in Fig. 4c. **Supplemental Fig. S7.** Effects of intermittent hypoxia on HIF-1α/C-EBP-β/ABC transporter axis and chemo-immuno-resistance in NCI-H1563 cells. NCI-H1563 cells were cultured in the following conditions: normoxia (at 20% O2 for 24 h, N), hypoxia (at 1% O2 for 24 h, H), hypoxia/normoxia (12 h at 1% O2 followed by 12 h at 20% O2, H/N), hypoxia/normoxia/hypoxia or intermittent hypoxia (12 h at 1% O2 followed by 12 h at 20% O2 and 12 h at 1% O2, H/N/H). a. Immunoblot of HIF-1α in whole cell extracts. Actin is included as control of equal protein loading. The image is representative of 1 out of 3 experiments. b. EPO1, VEGFA, GLUT1, ABCB1, ABCC1, ABCA1 mRNAs, measured by RT-PCR, in technical triplicates. Data are means±SD (*n* = 4 biological replicates). *p < 0.05,**p < 0.01,***p < 0.001: H, H/N, H/N/H versus N cells; °°°*p* < 0.001: H/N cells versus H cells. c. RNA-IP with an anti-HIF-1α antibody, followed by RT-PCR amplification (in technical triplicates) with primers for C/EBP-β LAP (upper panel) or LIP (lower panel) isoforms. Data are means±SD (*n* = 4 biological replicates). *p < 0.05,**p < 0.01,***p < 0.001: H, H/N, H/N/H versus N cells. d. Immunoblot of C/EBP-β LAP in whole cell extracts. Actin is included as control of equal protein loading. The image is representative of 1 out of 3 experiments. e. ChIP of C/EBP-β on ABCB1, ABCC1 and ABCA1 promoters, in technical triplicates. Data are means±SD (*n* = 4 biological replicates). *p < 0.05,**p < 0.01,***p < 0.001: H, H/N, H/N/H versus N cells. f. After normoxic and hypoxic cultures, cells were treated for 48 h in normoxia (20% O2) with increasing concentrations (from 1 × 10− 9 to 1 × 10− 5 M) of cisplatin (Pt) and docetaxel (Dx). Cell viability was measured by a chemiluminescence-based assay, in technical quadruplicates (*n* = 3 biological replicates). Representative [inhibitor] vs. normalized dose-response curves and relative IC50, obtained with the GraphPad Prism 9 software. g. Amount of released [14C]-IPP, considered an index of efflux, measured by liquid scintillation, in technical triplicates. Data are means±SD (*n* = 3 biological replicates). ***p < 0.001: H, H/N, H/N/H versus N cells. h. Percentage of Ki67+IFN-γ+ Vγ9Vδ2 T cells collected after the co-cultures with the NCI-H1563 cells, evaluated by flow cytometry, in technical duplicates. Data are means±SD (*n* = 5 biological replicates). *p < 0.05,***p < 0.001: H, H/N, H/N/H versus N cells. i. Percentage of annexin V+PI+ NCI-H1563 cells, as index of tumor cells killed by Vγ9Vδ2 T-cells, evaluated by flow cytometry, in technical duplicates. Data are means±SD (*n* = 5 biological replicates). ***p < 0.001: H, H/N, H/N/H versus N cells. **Supplemental Fig. S8.** Antioxidants defenses in NCI-H2228 cells subjected to hypoxia. NCI-H2228 cells were cultured in the following conditions: normoxia (at 20% O2 for 24 h, N), hypoxia (at 1% O2 for 24 h, H), hypoxia/normoxia (12 h at 1% O2 followed by 12 h at 20% O2, H/N), hypoxia/normoxia/hypoxia or intermittent hypoxia (12 h at 1% O2 followed by 12 h at 20% O2 and 12 h at 1% O2, H/N/H). a-e. Superoxide dismutase 2 (SOD2) and superoxide dismutase 1 (SOD1) activity (a-b), catalase (c) activity, GSH/GSSG ratio (d) and glutathione peroxidase (GPX) activity (e) were measured spectrophotometrically, in technical triplicates. Data are means±SD (*n* = 3 biological replicates). *p < 0.05,**p < 0.01,***p < 0.001: H, H/N, H/N/H cells versus N cells. f. Cell viability was measured after the different culture conditions indicated, by a chemiluminescence-based assay, in technical quadruplicates (*n* = 3 biological replicates). **Supplemental Fig. S9.** Effects of elesclomol and mitoQ on mitochondrial ROS. NCI-H2228 cells were cultured in the following conditions: normoxia (at 20% O2 for 24 h, N), hypoxia (at 1% O2 for24 h, H), hypoxia/normoxia (12 h at 1% O2 followed by 12 h at 20% O2, H/N), hypoxia/rnormoxia/hypoxia or intermittent hypoxia (12 h at 1% O2 followed by 12 h at 20% O2 and 12 h at 1% O2, H/N/H). Normoxic cells were treated with or without (−) 5, 10, 50 μM elesclomol (Es, a), hypoxic cells were treated with or without (−) 0.1, 0.2, 0.4 μM mitoquinol (mQ, b). Mitochondrial ROS, measured spectrofluorimetrically, in technical triplicates. Data are means±SD (*n* = 3 biological replicates). *p < 0.05,**p < 0.01,***p < 0.001: H, H/N, H/N/H cells or Es/−mQ-treated cells versus untreated N cells; °°*p* < 0.01,°°°*p* < 0.001: mQ-treated cells versus respective H, H/N, H/N/H untreated cells. **Supplemental Fig. S10.** Scavenging mitochondrial ROS sensitizes non-small cell lung cancer cells to docetaxel. NCI-H2228 cells were cultured in the following conditions: normoxia (at 20% O2 for 24 h, N), hypoxia (at 1% O2 for 24 h, H), hypoxia/normoxia (12 h at 1% O2 followed by 12 h at 20% O2, H/N), hypoxia/rnormoxia/hypoxia or intermittent hypoxia (12 h at 1% O2 followed by 12 h at 20% O2 and 12 h at 1% O2, H/N/H). After these incubation times, cells were treated for 48 h in normoxia (20% O2) with increasing concentrations (from 1 10− 9 to 1 × 10− 5 M) of docetaxel (Dx). When indicated, 0.4 μM mQ was added. Cell viability was measured by a chemiluminescence-based assay, in technical quadruplicates (*n* = 3 biological replicates). Representative (inhibitor) vs. normalized dose-response curves and relative IC50, obtained with the GraphPad Prism 9 software. **Supplemental Fig. S11.** Chemo-immuno-sensitizing effects of mitoquinol in NCI-H1563 cells grown in intermittent hypoxia. NCI-H1563 cells were cultured in the following conditions: normoxia (at 20% O2 for 24 h, N), hypoxia (at 1% O2 for 24 h, H), hypoxia/normoxia (12 h at 1% O2 followed by 12 h at 20% O2, H/N), hypoxia/normoxia/hypoxia or intermittent hypoxia (12 h at 1% O2 followed by 12 h at 20% O2 and 12 h at 1% O2, H/N/H). a. Mitochondrial ROS, measured spectrofluorimetrically, in technical triplicates. Data are means±SD (*n* = 4 biological replicates). ***p < 0.001: H, H/N/H versus N cells; °°°*p* < 0.001: H/N cells versus H cells. b. Hypoxic cells were treated with or without (−) 0.1, 0.2, 0.4 μM mitoquinol (mQ). Normoxic cells were included as internal control. Mitochondrial ROS were measured spectrofluorimetrically, in technical quadruplicates. Data are means±SD (*n* = 3 biological replicates). **p < 0.01,***p < 0.001:H, H/N, H/N/H cells or mQ-treated cells versus untreated N cells; °*p* < 0.05,°°°*p* < 0.001 mQ-treated cells versus respective H, H/N, H/N/H untreated cells. c. After normoxic and hypoxic cultures, cells were treated for 48 h in normoxia (20% O2) with increasing concentrations (from 1 × 10− 9 to 1 × 10− 5 M) of cisplatin (Pt, upper panels) and docetaxel (Dx, lower panels). When indicated, 0.4 μM mQ was added. Cell viability was measured by a chemiluminescence-based assay, in technical quadruplicates (*n* = 3 biological replicates). Representative [inhibitor] vs. normalized dose-response curves and relative IC50, obtained with the GraphPad Prism 9 software. d. Percentage of Ki67+IFN-γ+ Vγ9Vδ2 T cells collected after the co-cultures with the NCI-H1563 cells, evaluated by flow cytometry, in technical duplicates. Data are means±SD (*n* = 5 biological replicates). *p < 0.05,***p < 0.001: H, H/N, H/N/H versus N cells; °*p* < 0.05,°°°*p* < 0.001 mQ-treated cells versus respective H, H/N, H/N/H untreated cells. e. Percentage of annexin V+PI+ NCI-H1563 cells, as index of tumor cells killed by Vγ9Vδ2 T-cells, evaluated by flow cytometry, in technical duplicates. Data are means±SD (*n* = 5 biological replicates). ***p < 0.001: H, H/N, H/N/H versus N cells; °°°*p* < 0.001 mQ-treated cells versus respective H, H/N, H/N/H untreated cells. **Supplemental Fig. S12.** Dose-response effects of cisplatin and mitoquinol combination in LAP-overexpression NCI-H2228 tumors. 1 × 106 C/EBP-β LAP-overexpressing cells were injected subcutaneously in Hu-CD34+NSG mice. When tumor reached the volume of 50 mm3, animals (*n* = 4/group) were randomized and treated for 6 weeks as it follows: vehicle (0) group, treated with 0.1 ml saline solution intravenously (i.v.), once a week; mitoquinol (mQ) groups, treated with 10, 25, 50, 100 or 200 mg/kg daily via oral gavage, in the absence (−) or in the presence (+) of 2 mg/kg cisplatin (PT) i.v., once a week. To induce LAP intratumourally, 1 mg/ml doxycycline was added daily to the drinking water. Tumor growth was monitored by caliper. ***p < 0.001: PT + mQ-groups vs. PT-group (weeks 2–7). **Supplemental Fig. S13.** Tumors and post-mortem tissues characterization. 1 × 106 C/EBP-β LAP-overexpressing cells were injected subcutaneously in Hu-CD34+NSG mice. When tumor reached the volume of 50 mm3, animals (*n* = 6/group) were randomized and treated for 5 weeks as it follows: 1) vehicle (ctrl) group, treated with 0.1 ml saline solution intravenously (i.v.), once a week; 2) cisplatin (PT) group, treated with 2 mg/kg cisplatin i.v., once a week; 3) mitoquinol (mQ) group, treated with 100 μg/kg daily via oral gavage; 4) cisplatin + mitoquinol (PT + mQ) group, treated with 2 mg/kg cisplatin i.v., once a week and 100 μg/kg mitoquinol daily via oral gavage. To induce LAP intratumourally, 1 mg/ml doxycycline was added daily to the drinking water (LAP+ mice). a. Weight of the excised tumors. Data are means±SD (*n* = 6 tumors). ***p < 0.001:LAP+PT + mQ-group vs. LAP−ctrl-group; °°°*p* < 0.001:LAP+PT + mQ-group vs LAP+ctrl-group; ###*p* < 0.001:LAP+PT + mQ-group vs LAP+PT-group. b. Immunohistochemical analysis quantification. The amount of cells positive for C/EBP-β LAP, ABCB1, ABCB1, ABCA1 and cleaved caspase 3 was calculated counting 200 ± 25 cells/field, analyzing 5 fields for each treatment group, derived from each tumor and using Photoshop program. ***p < 0.001:LAP−PT-group vs LAP−ctrl-group, LAP+ctrl/PT-group vs. LAP−ctrl-group; °°°*p* < 0.001: LAP+mQ/PT + mQ-group vs LAP+ctrl-group; ###*p* < 0.001: LAP+mQ/PT + mQ-group vs LAP+PT-group. c. Animals weight were monitored weekly. d. Representative hematoxylin-eosin staining of liver, kidneys and spleen examined post-mortem. For each experimental group a minimum of 5 field were examined. Liver, kidneys: 63× objective, 20× ocular, bar: 50 μm; spleen: 63× objective, 10× ocular, bar: 100 μm. **Table S4.** Hematochemical parameters of treated animals.

## Data Availability

All data generated or analysed during this study are included in this published article and its supplemental information files.

## Abbreviations

pO_2_: O_2_ pressure
HIF-α: Hypoxia induced factor α
PHD: Prolyl hydroxylase domain
GLUT1: Glucose transporter 1
OXPHOS: Oxidative phosphorylation
ABC: ATP Binding Cassette
ABCB1/Pgp: ABC transporter B1/P-glycoprotein
ABCC1/MRP1: ABC transporter C1/multidrug resistance related protein 1
ER: Endoplasmic reticulum
C/EBP: CCAAT/Enhancer Binding Protein
ABCA1: ABC transporter A1
IPP: Isopentenyl pyrophosphate
NSCLC: Non-small cell lung cancer
mtROS: Mitochondrial reactive oxygen species
FBS: Featl bovine serum
mitoQ: [10-(2,5-dihydroxy-3,4-dimethoxy-6,ethylphenyl)decyl]triphenyl-phosphonium, monomethanesulfonate or mitoquinol
RLUs: Relative luminescence units
EPD: Eukariotic Promoter Database
RNA-IP: RNA-immunoprecipitation
HRE: Hypoxia-response element
ChIP: Chromatin immunoprecipitation
GFP: Green fluorescence protein
PBMC: Peripheral blood mononuclear cells
FITC: Fluorescein isothiocyanate
APC: Allophycocyanin
ETC: Electron transport chain
RFUs: Relative fluorescence units
CM-H_2_DCFDA: 5-(and-6)-chloromethyl-2′,7′-dichlorodihydro-fluorescein diacetate
UPN: Unknown patient number
PFS: Progression free survival
OS: Overall survival
FFPE: Formalin-fixed paraffin-embedded
s.c.: Subcutaneously
i.v.: Intravenously
RBC: Red blood cells
WBC: White blood cells
Hb: Haemoglobin
PLT: Platelets
LDH: Lactate dehydrogenase
AST: Aspartate aminotransferase
ALT: Alanine aminotransferase
AP: Alkaline phosphatase
CPK: Creatine phosphokinase
ANOVA: One-way analysis of variance
EPO1: Erythropoietin
VEGFA: Vascular endothelial growth factor A
