# Supplementary material for: Mitochondrial ROS drive resistance to chemotherapy and immune-killing in hypoxic non-small cell lung cancer
Source: J Exp Clin Cancer Res. 2022 Aug 11;41:243. doi: 10.1186/s13046-022-02447-6 (PMC9373288; doi:10.1186/s13046-022-02447-6)
Supplement: Supplementary file 1 — Additional file 1: Table S1. RT-PCR primers list. Table S2. Transcription factors binding ABCB1, ABCC1 and ABCA1 promoters. Table S3. Patient clinical-pathological features. Fig. S1. Intermittent hypoxia increases the IC50 of cisplatin and docetaxel in non-small cell lung cancer NCI-H2228 cells. NCI-H2228 cells were cultured in the following conditions: normoxia (at 20% O2 for 24 h, N), hypoxia (at 1% O2 for 24 h, H), hypoxia/normoxia (12 h at 1% O2 followed by 12 h at 20% O2, H/N), hypoxia/normoxia/hypoxia or intermittent hypoxia (12 h at 1% O2 followed by 12 h at 20% O2 and 12 h at 1% O2, H/N/H), then incubated for 48 h in normoxia (20% O2) with increasing concentrations (from 1 × 10− 9 to 1 × 10− 5 M) of cisplatin (Pt, a) and docetaxel (Dx, b). Cell viability was measured by a chemiluminescence-based assay, in technical quadruplicates (n = 4 biological replicates). Representative [inhibitor] vs. normalized dose-response curves and relative IC50, obtained with the GraphPad Prism 9 software. Fig. S2. Effects of intermittent hypoxia on ABC transporters expression. NCI-H2228 cells were cultured in the following conditions: normoxia (at 20% O2 for 24 h, N), hypoxia (at 1% O2 for 24 h, H), hypoxia/normoxia (12 h at 1% O2 followed by 12 h at 20% O2, H/N), hypoxia/normoxia/hypoxia or intermittent hypoxia (12 h at 1% O2 followed by 12 h at 20% O2 and 12 h at 1% O2, H/N/H). The expression level of the indicated ABC transporters was measured by a RT-PCR array and is represented in a colorimetric scale. The expression of each ABC transporter in normoxic cells was considered 1; the relative expression in the other experimental conditions is indicated in the heatmap. Supplemental Fig. S3. Overexpression of C/EBP-β LAP and LIP. Normoxic NCI-H2228 cells, wild-type (wt), overexpressing C/EBP-β LAP (LAP+) or overexpressing C/EBP-β LIP (LIP+) were lysed and immunoblotted with an anti-C/EBP-β antibody, recognizing both LAP and LIP isoforms. Actin is included as control of equal prot [file 13046_2022_2447_MOESM1_ESM.pdf]

Supplemental materials

Suppplemental Table S1. RT-PCR primers list

**Gene**	**Forward primer (5’-> 3’)**	**Reverse primer (5’-> 3’)**
EPO1	CAGACTTCTACGGCTTGCTG	GCTGAACACTGCAGCTTGAA
VEGFA	ATCTTCAAGCCATCCTGTGTGC	GCTCACCGCCTCGGCTTGT
GLUT1	CCTGCAGTTTGGCTACAACA	TAACGAAAAGGCCCACAGAG
ABCB1	TGCTGGAGCGGTTCTACG	ATAGGCAATGTTCTCAGCAATG
ABCC1	CATTCAGCTCGTCTTGTCCTG	GGATTAGGGTCGTGGATGGTT
ABCA1	CAGAGCTCACAGCAGGGAC	CTTCTCCGGAAGGCTTGTC-
HIF-1α	TGGTGCATCTCGAGACTTT	GAAGACATCGCGGGGAC
C/EBP-β LAP	ACCTCTTCTCCGACGACTAC	TCCTCCTTCCGCTTGCAG
C/EBP-β LIP	TGCAATCGGTTTAAACATGGC	ACAGACGCCTCTTTTCTCATAG
ABCB1 pro	GGCTAAACTTCTCAACTCTGGC	TGCTAAGGTGTAGGACGTCT
ABCC1 pro	TCATGTCTCCAGGCTTCAGT	GAGATTCAAACCCGTGAGCA
ABCA1 pro	AGGTGTCGGTAGCATTTTCTTG	TGAGCCAAGATTGTGCCACT
Unspecific primers (ChIP)	GTGGTGCCTGAGGAAGAGAG	GCAACAAGTAGGCACAGCA
S14	CGAGGCTGATGACCTGTTCT	GCCCTCTCCCACTCTCTCTT

pro: promoter; ChIP: chromatin immunoprecipitation

Supplemental Table S2. Transcription factors binding *ABCB1*, *ABCC1* and *ABCA1* promoters

**ABCB1**	**ABCC1**	**ABCA1**
AhR:Arnt	AhR:Arnt	AP-1
AP-1	AP-2αA	AP-2αA
AP-2αA	AR	ATF3
AR	ATF-1	C/EBPα
ATF3	ATF3	C/EBPβ
C/EBPα	C/EBPβ	c-Ets-1
C/EBPα	c-Ets-1	c-Ets-2
c-Ets-1	c-Ets-2	c-Jun
c-Ets-2	c-Jun	c-Myb
c-Jun	c-Myb	c-Myc
c-Myb	E2F-1	E2F-1
COUP-TF1	EBF	Elk-1
CTF	Elk-1	ENKTF-1
E2F-1	ENKTF-1	ER-α
Elk-1	ER-α	ETF
ENKTF-1	ETF	FOXP3
ETF	FOXP3	GATA-1
FOXP3	GATA-1	GCF
GATA-1	GATA-2	GR
GATA-2	GCF	GR-α
GCF	GR	GR-β
GR	GR-α	HIF-1
GR-α	GR-β	HNF-1B
GR-β	HNF-1A	HNF-1C
HNF-1A	HNF-1B	HNF-3α
HNF-1B	HNF-1C	IRF-1
HNF-1C	HNF-3α	LEF-1
HNF-3α	Ik-1	MAZ
HNF-4α	IRF-1	NF-1
HOXD10	IRF-2	NF-AT1
HOXD9	LEF-1	NF-AT2
IRF-1	MEF-2A	NFI/CTF

LEF-1	NF-1	NF-kB1
NF-1	NF-AT1	NF-Y
NF-AT1	NF-AT2	p53
NF-AT2	NFI/CTF	Pax-5
NFI/CTF	NF-kB	POU2F1
NF-Y	p53	PR A
p53	Pax-5	PR B
Pax-5	PEA3	PXR-1:RXR-α
PEA3	PR A	RAR-β
POU2F1	PR B	RelA
POU2F2	PXR-1:RXR-α	RXR-α
PR A	RAR-α1	Sp1
PR B	RAR-β	SRF
PXR-1:RXR-α	RAR-β:RXR-α	SRY
RelA	RelA	STAT1β
SRY	RXR-α	STAT4
STAT1β	Sp1	TBP
STAT4	SRY	TCF-4
STAT5A	STAT4	TCF-4E
T3R-β1	T3R-β1	TFIID
TCF-4	TCF-4E	TFII-I
TCF-4E	TFIID	USF1
TFIID	TFII-I	USF2
TFII-I	USF2	VDR
VDR	VDR	
WT1	WT1	
XBP-1	XBP-1	
YY1		

Transcription factors predicted to bind *ABCB1*, *ABCC1* and *ABCA*1 promoters (TRANSFAC software v 8.3)

Supplemental Table S3. Patient clinical-pathological features

UPN	Gender	Age	Smoking habits	Stage	C/EPB-β LAP Score	% positive cells	PFS (months)	OS (months)
1	M	63	Nsm	IV	++	40	14	34
2	M	71	CSm	III	+++	88	6	18
3	F	64	CSm	IV	+++	78	9	12
4	M	81	FSm	IV	+	12	17	24
5	M	55	FSm	IV	++	43	24	65
6	M	68	NSm	IV	+	7	4	18
7	F	67	CSm	IV	+	18	7	14
8	M	54	CSm	IV	+++	76	16	36
9	F	69	CSm	IV	++	34	16	43
10	F	76	CSm	IV	+++	89	4	7
11	M	71	FSm	III	+	14	23	45
12	M	55	FSm	IV	+++	89	3	6
13	F	83	n.a.	IV	++	56	11	24
14	M	77	FSm	III	+	21	15	34
15	F	64	NSm	IV	++	37	23	29
16	M	78	CSm	III	+++	75	11	18
17	M	79	CSm	IV	++	59	14	23
18	M	66	CSm	IV	+	13	22	47
19	F	53	CSm	IV	+++	89	8	15
20	M	59	FSm	IV	++	47	11	26
21	F	78	FSm	IV	+	24	19	31
22	M	68	CSm	III	++	39	15	28
23	M	64	NSm	IV	+	18	16	32
24	M	66	NSm	IV	++	46	11	24
25	M	69	FSm	IV	+++	79	3	5
26	F	67	FSm	IV	++	55	12	21
27	F	83	CSm	IV	+	27	15	35
28	F	80	CSm	IV	+++	93	9	18
29	M	73	CSm	III	++	42	7	15
30	M	72	FSm	IV	+	7	17	29
31	F	57	CSm	III	+++	76	8	13
32	M	59	CSm	IV	++	41	14	28
33	F	73	CSm	IV	+++	69	10	16
34	M	77	CSm	IV	+	22	14	23
35	F	64	NSm	IV	++	56	13	16
36	M	69	CSm	III	+++	72	8	14
37	M	58	CSm	IV	+	15	16	27
38	F	67	FSm	III	++	61	13	22
39	M	74	NSm	IV	+++	89	8	16
40	M	59	FSm	IV	+	16	14	23

41	M	79	CSm	IV	+	25	9	25
42	F	78	CSm	IV	++	37	12	14
43	F	78	FSm	III	++	51	14	25
44	M	68	NSm	IV	+++	78	2	11
45	M	65	FSm	III	+	19	14	26
46	F	69	CSm	III	+++	89	7	16
47	F	74	CSm	IV	+	24	15	42
48	F	75	CSm	IV	+++	82	11	14
49	F	65	FSm	IV	++	56	15	23
50	M	71	CSm	IV	+	11	16	28
51	M	73	NSm	III	+	19	18	34
52	M	59	CSm	IV	+++	75	6	13
53	F	65	CSm	IV	++	63	13	22
54	M	66	FSm	III	++	46	14	17
55	M	69	CSm	III	+	21	14	29
56	F	80	CSm	IV	+++	88	5	27
57	M	67	CSm	IV	++	37	13	21
58	F	69	FSm	IV	+++	76	8	11
59	M	76	NSm	IV	++	44	7	15
60	M	71	CSm	IV	++	53	8	20

UPN: unknown patient number; F: female; M: male; NSm: never smoker; CSm: current smoker; FSm: former smoker; PFS: progression free survival; OS: overall survival.

Supplemental Figure S1

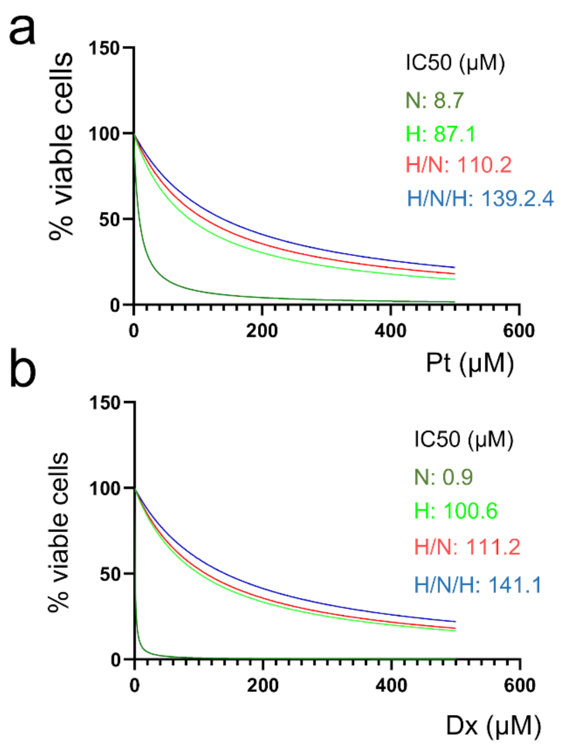

Supplemental Figure S1. Intermittent hypoxia increases the IC_{50} to cisplatin and docetaxel in non-small cell lung cancer NCI-H2228 cells

NCI-H2228 cells were cultured in the following conditions: normoxia (at 20% O_2 for 24h, N), hypoxia (at 1% O_2 for 24h, H), hypoxia/normoxia (12h at 1% O_2 followed by 12h at 20% O_2, H/N), hypoxia/normoxia/hypoxia or intermittent hypoxia (12h at 1% O_2 followed by 12h at 20% O_2 and 12h at 1% O_2, H/N/H), then incubated for 48h in normoxia (20% O_2) with increasing concentrations (from 1×10^{-9} to 1×10^{-5}M) of cisplatin (Pt, **a**) and docetaxel (Dx, **b**). Cell viability was measured by a chemiluminescence-based assay, in technical quadruplicates (n=4 biological replicates). Representative [inhibitor] vs. normalized dose-response curves and relative IC_{50}, obtained with the GraphPad Prism 9 software.

Supplemental Figure S2

	N	H	H/N	H/N/H
ABCA1	1	0.41	0.56	0.61
ABCA2	1	2.1	2.3	2.1
ABCA3	1	2.1	1.2	2.1
ABCA4	1	1.3	1.2	1.4
ABCA9	1	2.3	2	2.3
ABCA12	1	2.1	2	2.3
ABCA13	1	2.8	2.4	2.4
ABCB1	1	8.9	13.4	17.5
ABCB2	1	2.1	2.7	1.3
ABCB3	1	1.3	1.6	1.3
ABCB4	1	2.4	1.4	1.9
ABCB5	1	1.2	1.7	0.9
ABCB6	1	1.7	1.2	1.4
ABCB11	1	1.5	1.3	1.2
ABCC1	1	6.4	10.3	21.9
ABCC2	1	2.3	1.3	1.4
ABCC3	1	1.2	1.5	1.3
ABCC4	1	1.6	1.4	1.4
ABCC5	1	1.9	2.3	2.8
ABCC6	1	3.2	3.9	2.1
ABCC11	1	1.3	1.8	1.2
ABCC12	1	1.4	1.8	1.3
ABCC19	1	3.2	3.1	2.7
ABCD1	1	2.1	2.9	3.4
ABCD3	1	2.1	2.8	2.1
ABCD4	1	2.9	1.4	1.2
ABCF1	1	2.7	2.4	3.7
ABCG2	1	2.1	3.4	3.2
ABCG8	1	2	2.3	2.1

Supplemental Figure S2. Effects of intermittent hypoxia on ABC transporters expression

NCI-H2228 cells were cultured in the following conditions: normoxia (at 20% O_2 for 24h, N), hypoxia (at 1% O_2 for 24h, H), hypoxia/normoxia (12h at 1% O_2 followed by 12h at 20% O_2, H/N), hypoxia/normoxia/hypoxia or intermittent hypoxia (12h at 1% O_2 followed by 12h at 20% O_2 and 12h at 1% O_2, H/N/H). The expression level of the indicated ABC transporters was measured by a RT-PCR array and is represented in a colorimetric scale. The expression of each ABC transporter in normoxic cells was considered 1; the relative expression in the other experimental conditions is indicated in the heatmap.

Supplemental Figure S3

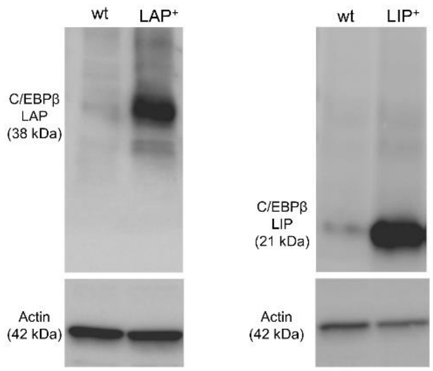

Supplemental Figure S3. Overexpression of C/EBP-β LAP and LIP

Normoxic NCI-H2228 cells, wild-type (wt), overexpressing C/EBP-β LAP (LAP^+) or overexpressing C/EBP-β LIP (LIP^+) were lysed and immunoblotted with an anti-C/EBP-β antibody, recognizing both LAP and LIP isoforms. Actin is included as control of equal protein loading. The image is representative of 1 out of 3 experiments.

Supplemental Figure S4

a

C/EBP βLAP RNA (CEBPB b transcript; NM_001285878); 2113 nt

TCCCAATCCCGGGGCGGCCGGGCGGGGGTGGGCAGGGGGCGTGAGGCCGCCCCTGCGTCCCGGGGGCCCCC
CGAAAACGCGCTCCGGGTGCCCGGTCCCTCCGCTGCGCCCTGCCGCCGTCCTCCCGGGGGTCTCGGGCGGCC
GCGGCCGTGTCCTTCGCGTCCCGGCGGCGCGGCGGGAGGGGCCGGCGTGACGCAGCGGTTGCTACGGGCCG
CCCTTATAAATAACCGGGCTCAGGAGAAACTTTAGCGAGTCAGAGCCGCGCACGGGACTGGGAAGGGGACCCAC
CCGAGGGTCCAGCCACCAGCCCCCTCACTAATAGCGGCCACCCCGGCAGCGGCGGCAGCAGCAGCAGCGACG
CAGCGGCGACAGCTCAGAGCAGGGAGGCCGCGCCACCTGCGGGCCGGCCGGAGCGGGCAGCCCCAGGCCCC
CTCCCCGGGCACCCGCGTTCATGCAACGCCTGGTGGCCTGGGACCCAGCATGTCTCCCCCTGCCGCCGCCGCC
GCCTGCCTTTAAATCCATGGAAGTGGCCAACTTCTACTACGAGGCGGACTGCTTGGCTGCTGCGTACGGCGGCA
AGGCGGCCCCCGCGGCGCCCCCCGCGGCCAGACCCGGGCCGCGCCCCCCCGCCGGCGAGCTGGGCAGCATC
GGCGACCACGAGCGCGCCATCGACTTCAGCCCGTACCTGGAGCCGCTGGGCGCGCCGCAGGCCCCGGCGCCC
GCCACGGCCACGGACACCTTCGAGGCGGCTCCGCCCGCGCCCGCCCCCGCGCCCGCCTCCTCCGGGCAGCAC
CACGACTTCCTCTCCGACCTCTTCTCCGACGACTACGGGGGCAAGAACTGCAAGAAGCCGGCCGAGTACGGCTA
CGTGAGCCTGGGGCGCCTGGGGGCCGCCAAGGGCGCGCTGCACCCCGGCTGCTTCGCGCCCCTGCACCCACC
GCCCCCGCCGCCGCCGCCGCCCGCCGAGCTCAAGGCGGAGCCGGGCTTCGAGCCCGCGGACTGCAAGCGGAA
GGAGGAGGCCGGGGCGCCGGGCGGCGGCGCAGGCATGGCGGCGGGCTTCCCGTACGCGCTGCGCGCTTACC
TCGGCTACCAGGCGGTGCCGAGCGGCAGCAGCGGGAGCCTCTCCACGTCCTCCTCGTCCAGCCCGCCCGGCAC
GCCGAGCCCCGCTGACGCCAAGGCGCCCCCGACCGCCTGCTACGCGGGGGCCGCGCCGGCGCCCTCGCAGGT
CAAGAGCAAGGCCAAGAAGACCGTGGACAAGCACAGCGACGAGTACAAGATCCGGCGCGAGCGCAACAACATC
GCCGTGCGCAAGAGCCGCGACAAGGCCAAGATGCGCAACCTGGAGACGCAGCACAAGGTCCTGGAGCTCACGG
CCGAGAACGAGCGGCTGCAGAAGAAGGTGGAGCAGCTGTCGCGCGAGCTCAGCACCCTGCGGAACTTGTTCAA
GCAGCTGCCCGAGCCCCTGCTCGCCTCCTCCGGCCACTGCTAGCGCGGCCCCCGCGCGCGTCCCCCTGCCGG
CCGGGGCTGAGACTCCGGGGAGCGCCCGCGCCCGCGCCCTCGCCCCCGCCCCCGGCGGCGCCGGCAAAACTT
TGGCACTGGGGCACTTGGCAGCGCGGGGAGCCCGTCGGTAATTTTAATATTTTATTATATATATATATCTATATTTT
TGTCCAAACCAACCGCACATGCAGATGGGGCTCCCGCCCGTGGTGTTATTTAAAGAAGAAACGTCTATGTGTACA
GATGAATGATAAACTCTCTGCTTCTCCCTCTGCCCCTCTCCAGGCGCCGGCGGGCGGGCCGGTTTCGAAGTTGA
TGCAATCGGTTTAAACATGGCTGAACGCGTGTGTACACGGGACTGACGCAACCCACGTGTAACTGTCAGCCGGG
CCCTGAGTAATCGCTTAAAGATGTTCCTACGGGCTTGTTGCTGTTGATGTTTTGTTTTGTTTTGTTTTTTGGTCTTTT
TTTGTATTATAAAAAATAATCTATTTCTATGAGAAAAGAGGCGTCTGTATATTTTGGGAATCTTTTCCGTTTCAAGCA
TTAAGAACACTTTTAATAAACTTTTTTTTGAGAATGGTTACAAAGCCTTTTGGGGGCAGTAAAAAAA

b

C/EBP β LIP RNA (CEBP C transcript; NM_001285879); 1068 nt

ATGGCGGCGGGCTTCCCGTACGCGCTGCGCGCTTACCTCGGCTACCAGGCGGTGCCGAGCGGCAGCAGCGGG
AGCCTCTCCACGTCCTCCTCGTCCAGCCCGCCCGGCACGCCGAGCCCCGCTGACGCCAAGGCGCCCCCGACC
GCCTGCTACGCGGGGGCCGCGCCGGCGCCCTCGCAGGTCAAGAGCAAGGCCAAGAAGACCGTGGACAAGCAC
AGCGACGAGTACAAGATCCGGCGCGAGCGCAACAACATCGCCGTGCGCAAGAGCCGCGACAAGGCCAAGATGC
GCAACCTGGAGACGCAGCACAAGGTCCTGGAGCTCACGGCCGAGAACGAGCGGCTGCAGAAGAAGGTGGAGC
AGCTGTCGCGCGAGCTCAGCACCCTGCGGAACTTGTTCAAGCAGCTGCCCGAGCCCCTGCTCGCCTCCTCCGG
CCACTGCTAGCGCGGCCCCCGCGCGCGTCCCCCTGCCGGCCGGGGCTGAGACTCCGGGGAGCGCCCGCGCC
CGCGCCCTCGCCCCCGCCCCCGGCGGCGCCGGCAAAACTTTGGCACTGGGGCACTTGGCAGCGCGGGGAGCC
CGTCGGTAATTTTAATATTTTATTATATATATATATCTATATTTTTGTCCAAACCAACCGCACATGCAGATGGGGCT
CCCGCCCGTGGTGTTATTTAAAGAAGAAACGTCTATGTGTACAGATGAATGATAAACTCTCTGCTTCTCCCTCTGC
CCCTCTCCAGGCGCCGGCGGGCGGGCCGGTTTCGAAGTTGATGCAATCGGTTTAAACATGGCTGAACGCGTGT
GTACACGGGACTGACGCAACCCACGTGTAACTGTCAGCCGGGCCCTGAGTAATCGCTTAAAGATGTTCCTACGG
GCTTGTTGCTGTTGATGTTTTGTTTTGTTTTGTTTTTTGGTCTTTTTTTGTATTATAAAAAATAATCTATTTCTATGAG
AAAAGAGGCGTCTGTATATTTTGGGAATCTTTTCCGTTTCAAGCATTAAGAACACTTTTAATAAACTTTTTTTTGAGA
ATGGTTACAAAGCCTTTTGGGGGCAGTAAAAAAA

HRE

LIP ATG

Supplemental Figure S4. C/EBP-β mRNA sequence

Sequence of C/EBP-β LAP (**a**) and LIP (**b**) mRNA. The hypoxia-response element (HRE) is highlighted in cyano, the ATG codon of LIP mRNA in green.

Supplemental Figure S5

a *ABCB1* promoter (RefSeq NM_001348945)

GGTATATCCAGTGCATTGTTGGCACCGTGGGACCAGAAGGTAGTGACCCCCCTGGACCCAGCTTTCACTATCT
TGTGTGTGTCTATTATTTCTCAACCTGCCGATCCGCCTAAGAACAAAGAGAGAGCCCCGTTGCATTGCAGGCTG
CTGGCCAGATCCCACAATACATACACAGATTCCAAAATGCATTCTTAACTCTTAAAAGATTTTGGTTACTTATCAC
TATTCTGTCTACTTTTCTGTAATTGAGAAAAGTATTTGTCAACTCATTTTTTCTCTCTGTGACAGCTCAGTCATTTA
CAAAGTTTTATTTTATACTTTACTCCTTCCTTCAATTTGTGCTAAAACATTGTGAAAATTAACATTTCTTTGGAACA
CAACTTTTTCATTATAAATTAAATACTGATATAATTGCAAAGTAAACAAATGAATTTCCATAAAGCTAATTTATCTTT
ATATTTTCCATACTTATTACTTCAAATTCTTGTTACATTTTCAATTTTGTTTGAAATATCATATGGTATTTTAAATTG
AAATTTACTAATTATTTTTTAGCCAGTGGATAAAGAGAAATTAAAAATTTGAACAAATTAATTCAATTTTTACTTCA
CTTCTCATTTGAAGGTCTTCCCAGTAACCTACCAAAAGAAGTCTCTCTCTTTTTTTTTTTTTTTTTTTAGCTTAGATT
CTAATCTTCATTCTGCCTATTCTGGCTAAACTTCTCAACTCTGGCTATTTTTCAATTATACCCCAATCCCTAAGCC
ATGTAACTCTTCGAGGTTTTTGTTTGTTTTTTCAACTGCTCATTAAGACGTCCTACACCTTAGCAAAAAGATCACA
CATATTTCTTCAATGCTTTGGAGCCATAGTCATGTACTCAAAATTTATTTTATCTCTACTCCCACCCTTCCTCCAC
CCAAACTTATCCTTGGGTTTTCACTGATTAGTGCTTTCAAGCCTGCCTGCCTTAGTTCATGTAGCTCCTCCTCTG
GTACTGGGAT

b *ABCC1* promoter (RefSeq NM_004996)

CGGCTAATTTTTGTGTTTTTAGTAGAGACAGGGTTTTGCTATGTTGGCTAGGCTGGTCTTGAACTCCTGACCTT
ATGTGATCGGCCCGCCTCGGCTTCCCAAAGTGCTTGATTACAAGCACGAGCCACCATGCCTGGCCTACGATT
ATCATGTCTCCAGGCTTCAGTTTCCTTACCTCTGAGGTGAGGATCAATAACCTGGTTATTTCTAAGTGATGCTT
AAAGAGGTTGAGAAGAAGCAAAGGCGCTCTGCACACAGGTGAGGTCAACAGCTGTTAGTGATGGAGACTGGC
GCCGTCTGGGTCATCAGTGCTCCGGAATCAGGCTGCTCACGGGTTTGAATCTCACTTCCACTTTCACTCCTTA
TTAACTGTGTGACCTCGGGCAAGTCACTTCGCCTTTCTGAACCTCAGTTTCCCCATCTGTAAAAGGTAGGTGA
ATAACAGTATTCACCTCCTTCTGTGTGACTCAGCTTTGGAGTCAGCGGACCGGGTTCAAATCCCAGCCCTGCA
AGTGATTAGCCAGGTGACCCTGGGCAGAGGGAATCACTCAACCTCTCTGCACCTGGGTTTCTTCCTTCATGAA
CGTGGAGACTTTACAGGATGAAATGAGGGCACAGTTAAGGCGCCCGGTACACTCCAGGCAGGTAGGGGGCT
CCGTTCACGTTATTTTCCCCTGGTGACGGATACTGTCCTTAAACAGCATTTGAAAAGTGGTCGCAGGGTGTGT
GGCCCCAAAGATCCCAGGCGCTTCCGGAAGGCGAGCCAACGCTCCCCAGGCCCGTCCGCGAGCGGGTGGG
CCTAAGCCTTGGAGGATCTGGGGTGGGGGTGGCGCGGGGTCCAGGCCCGGGGCGCCGAGAGGTGGCTGG
TCCGGCTGCCCACGCCGAGACGCGCGAGGTGAGCGGGCGCCGGGGCGGGGCGGGGTGGGGCGGGGCCG
CCGCATCCCCGTGACGCGCGGGCCAACCAGGCGGCGTTGCGGCCCCGGCCCCGGCTCCCTGCGCCGCCG
CCGCCGCCGCCGCCAGCGCTAGCGCCAGCAGCCGGGCCCGATCACC

c *ABCA1* promoter (RefSeq NM_080282)

GAGAGCCAGGTGTCGGTAGCATTTTCTTGGTGTTTACTCACACTCATCTAAGGCACGTTGTGGTTTTCCAGATT
AGGAAACTGCTTTATTGATGGTGCTTTTTTTTTTTTTTTTTTGAGACAGAGTCTCGCTCTGTCGCCATGCTGGAGT
GTAGTGGCACAATCTTGGCTCACTGCACCTCCGCCTGCCAGGTTCAGCGATTCTCCTGCCTCAGCCTCCCAAG
TAGCTGGGACTACAGGTGCCTGCCACCATGCCCAGCTAATTTTTGTATTTTTAGTAGAGACGGGGTTTCACCG
TATTGGCTAGGATGGTCTCGATTTCTTGACCTCGTGATCCGCCTGCCTCGGCCTCCCAAAGTGCTGGGATTAT
AGGCTTGAGCCACCACGCCTGGCCGATGGTGCTTTTTATCATTTGAAGGACTCAGTTGTATAACCCACTGAAAA
TTAGTATGTAAGGAAGTTCAGGGAATAGTATAAGTCACTCCAGGCTTGAGGCAAAATTTACAAATGCTGCTGAC
TTTGTATGTAAGGGGAGGCATTTTCTTAGAAAAGAGAGGTAGGTCTCTGGGATTCCAGTATGCCATTTCCATCC
TCAGTGTTTTTGGCCACCTGAGAGAGGTCTATTTTCAGAAATGCATTCTTCATTCCCAGATGATAACATCTATAG
AACTAAAATGATTAGGACCATAACACGTAGCTCCTAGCCTGCTGTCGGAACACCTCCCGAGTCCCTCTTTGTG
GGTGAACCCAGAGGCTGGGAGCTGGTGACTCATGATCCATTGAGAAGCAGTCATGATGCAGAGCTGTGTGTT
GGAGGTCTCAGCTGAGAGGGCTGGATTAGCAGTCCTCATTGGTGTATGGCTTTGCAGCAATAACTGATGGCTG
TTTCCCCTCCTGCTTTATCTTTCAGTTAATGACCAGCCACGGCGTCCCTGCTGTGAGCTCTGGCCGCTGCCTT
CCAGGGCTCCCGAGCCACACGCTGGGGGTGCTGGCTGAGGGAAC

CAAT box

Supplemental Figure S5. *ABCB1*, *ABCC1* and *ABCA1* promoters sequence

Sequences of *ABCB1* (**a**), *ABCC1* (**b**) and ABCA1 (**c**) promoters. The putative binding sites for C/EBP-β (CAAT boxes) are highlighted in yellow.

Supplemental Figure S6

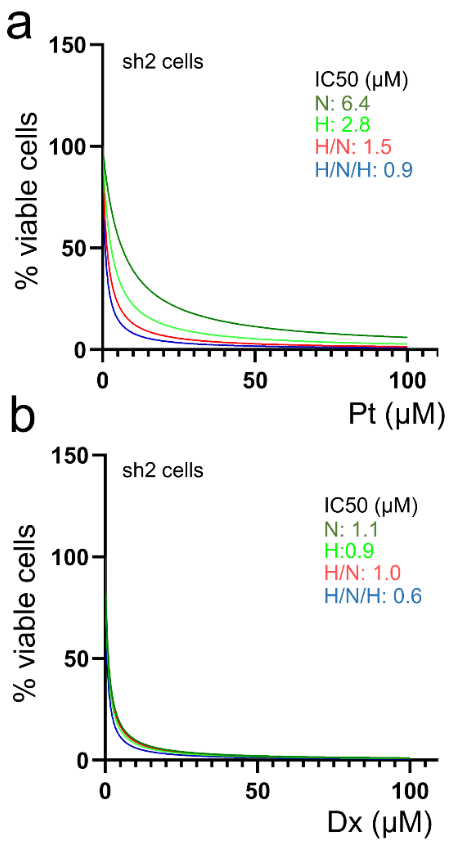

Supplemental Figure S6. Effects of C/EBP-β silencing on sensitivity to cisplatin and docetaxel

NCI-H2228 cells, stably silenced with the C/EBP-β-targeting sequence #2 (sh2), were cultured in the following conditions: normoxia (at 20% O_2 for 24h, N), hypoxia (at 1% O_2 for 24h, H), hypoxia/normoxia (12h at 1% O_2 followed by 12h at 20% O_2, H/N), hypoxia/normoxia/hypoxia or intermittent hypoxia (12h at 1% O_2 followed by 12h at 20% O_2 and 12h at 1% O_2, H/N/H). After normoxic and hypoxic cultures, cells were treated for 48h in normoxia (20% O_2) with increasing concentrations (from 1×10^{-9} to 1×10^{-5}M) of cisplatin (Pt, **a**) and docetaxel (Dx, **b**). Cell viability was measured by a chemiluminescence-based assay, in technical quadruplicates (n=3 biological replicates). Representative [inhibitor] vs. normalized dose-response curves and relative IC_{50}, obtained with the GraphPad Prism 9 software. The dose-response curves and relative IC_{50} in NCI-H2228 cells treated with a non-targeting sequence (scr) shRNA sequence are reported in Figure 4c.

Supplemental Figure S7

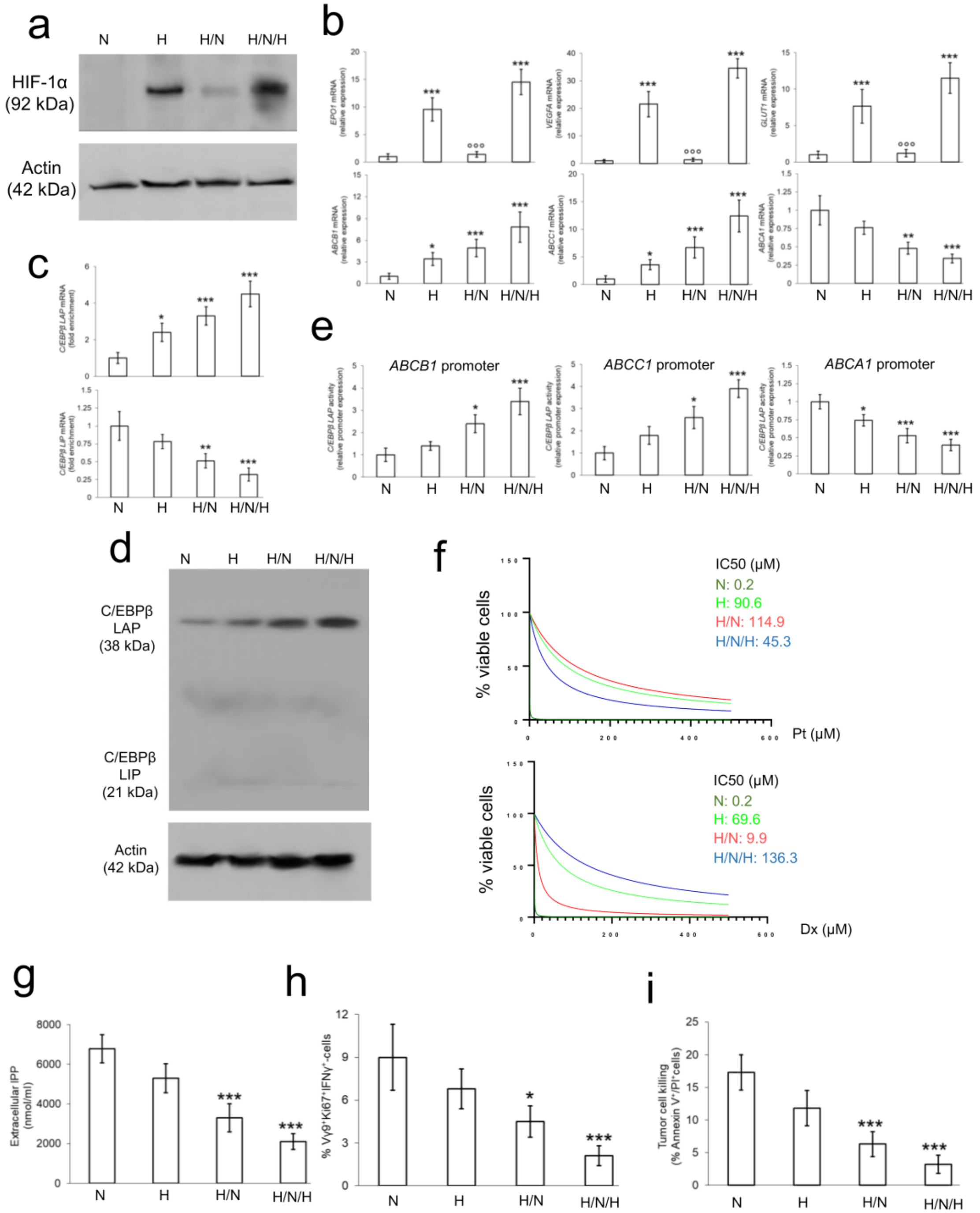

Supplemental Figure S7. Effects of intermittent hypoxia on HIF-1α/C-EBP-β/ABC transporter axis and chemo-immuno-resistance in NCI-H1563 cells

NCI-H1563 cells were cultured in the following conditions: normoxia (at 20% O_2 for 24h, N), hypoxia (at 1% O_2 for 24h, H), hypoxia/normoxia (12h at 1% O_2 followed by 12h at 20% O_2, H/N),

hypoxia/normoxia/hypoxia or intermittent hypoxia (12h at 1% O_2 followed by 12h at 20% O_2 and 12h at 1% O_2, H/N/H). **a.** Immunoblot of HIF-1α in whole cell extracts. Actin is included as control of equal protein loading. The image is representative of 1 out of 3 experiments. **b.** *EPO1*, *VEGFA*, *GLUT1*, *ABCB1*, *ABCC1*, *ABCA1* mRNAs, measured by RT-PCR, in technical triplicates. Data are means±SD (n=4 biological replicates). *p<0.05,**p<0.01,***p<0.001: H, H/N, H/N/H versus N cells; °°°p<0.001: H/N cells versus H cells. **c.** RNA-IP with an anti-HIF-1α antibody, followed by RT-PCR amplification (in technical triplicates) with primers for C/EBP-β LAP (upper panel) or LIP (lower panel) isoforms. Data are means±SD (n=4 biological replicates). *p<0.05,**p<0.01,***p<0.001: H, H/N, H/N/H versus N cells. **d.** Immunoblot of C/EBP-β LAP in whole cell extracts. Actin is included as control of equal protein loading. The image is representative of 1 out of 3 experiments. **e.** ChIP of C/EBP-β on *ABCB1*, *ABCC1* and *ABCA1* promoters, in technical triplicates. Data are means±SD (n=4 biological replicates). *p<0.05,**p<0.01,***p<0.001: H, H/N, H/N/H versus N cells. **f.** After normoxic and hypoxic cultures, cells were treated for 48h in normoxia (20% O_2) with increasing concentrations (from 1×10^{-9} to 1×10^{-5}M) of cisplatin (Pt) and docetaxel (Dx). Cell viability was measured by a chemiluminescence-based assay, in technical quadruplicates (n=3 biological replicates). Representative [inhibitor] vs. normalized dose-response curves and relative IC_{50}, obtained with the GraphPad Prism 9 software. **g.** Amount of released [^{14}C]-IPP, considered an index of efflux, measured by liquid scintillation, in technical triplicates. Data are means±SD (n=3 biological replicates). ***p<0.001: H, H/N, H/N/H versus N cells. **h.** Percentage of Ki67^{+}IFN-γ^{+} Vγ9Vδ2 T cells collected after the co-cultures with the NCI-H1563 cells, evaluated by flow cytometry, in technical duplicates. Data are means±SD (n=5 biological replicates). *p<0.05,***p<0.001: H, H/N, H/N/H versus N cells. **i.** Percentage of annexin V^{+}PI^{+} NCI-H1563 cells, as index of tumor cells killed by Vγ9Vδ2 T-cells, evaluated by flow cytometry, in technical duplicates. Data are means±SD (n=5 biological replicates). ***p<0.001: H, H/N, H/N/H versus N cells.

Supplemental Figure S8

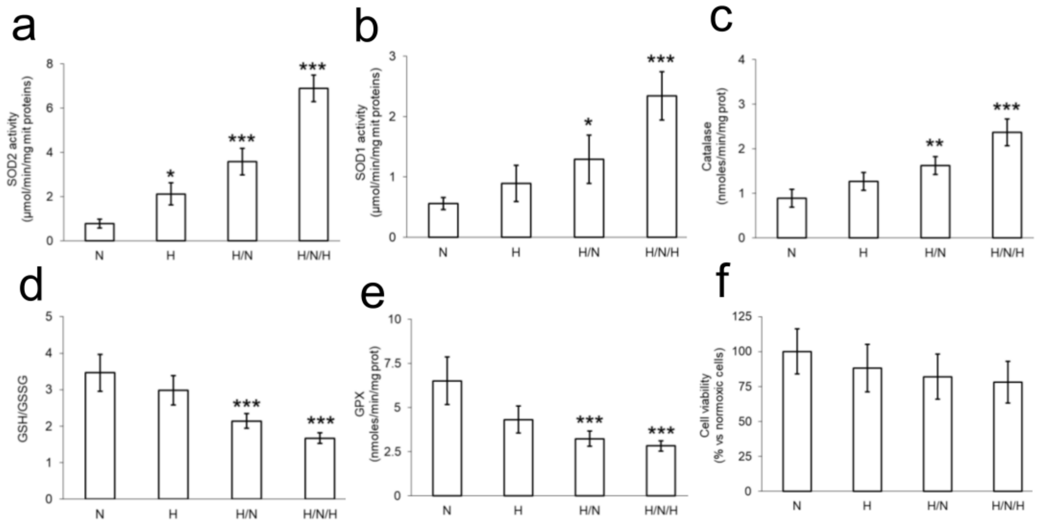

Supplemental Figure S8. Antioxidants defenses in NCI-H2228 cells subjected to hypoxia. NCI-H2228 cells were cultured in the following conditions: normoxia (at 20% O_2 for 24h, N), hypoxia (at 1% O_2 for 24h, H), hypoxia/normoxia (12h at 1% O_2 followed by 12h at 20% O_2, H/N), hypoxia/normoxia/hypoxia or intermittent hypoxia (12h at 1% O_2 followed by 12h at 20% O_2 and 12h at 1% O_2, H/N/H). **a-e.** Superoxide dismutase 2 (SOD2) and superoxide dismutase 1 (SOD1) activity (**a-b**), catalase (**c**) activity, GSH/GSSG ratio (**d**) and glutathione peroxidase (GPX) activity (**e**) were measured spectrophotometrically, in technical triplicates. Data are means±SD (n=3 biological replicates). *p<0.05,**p<0.01,***p<0.001: H, H/N, H/N/H cells versus N cells. **f.** Cell viability was measured after the different culture conditions indicated, by a chemiluminescence-based assay, in technical quadruplicates (n=3 biological replicates).

Supplemental Figure S9

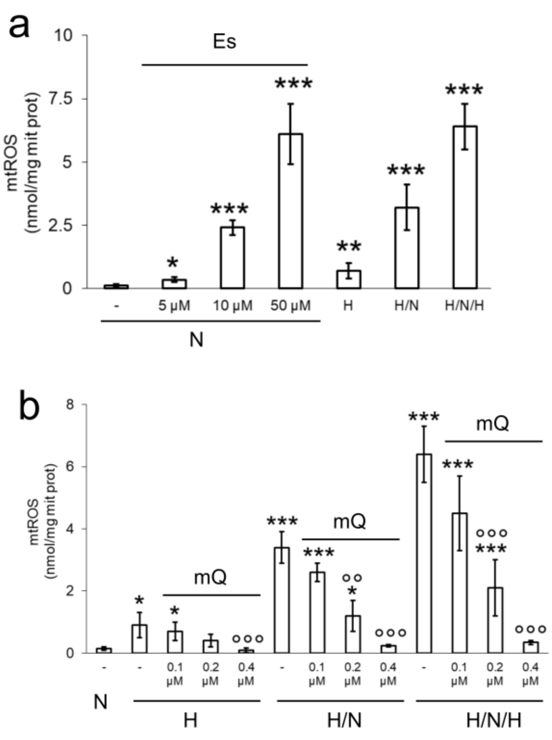

Supplemental Figure S9. Effects of elesclomol and mitoQ on mitochondrial ROS

NCI-H2228 cells were cultured in the following conditions: normoxia (at 20% O_2 for 24h, N), hypoxia (at 1% O_2 for24 h, H), hypoxia/normoxia (12h at 1% O_2 followed by 12h at 20% O_2, H/N), hypoxia/rnormoxia/hypoxia or intermittent hypoxia (12h at 1% O_2 followed by 12h at 20% O_2 and 12h at 1% O_2, H/N/H). Normoxic cells were treated with or without (-) 5, 10, 50µM elesclomol (Es, **a**), hypoxic cells were treated with or without (-) 0.1, 0.2, 0.4µM mitoquinol (mQ, **b**). Mitochondrial ROS, measured spectrofluorimetrically, in technical triplicates. Data are means±SD (n=3 biological replicates). *$p<0.05$,**$p<0.01$,***$p<0.001$: H, H/N, H/N/H cells or Es/-mQ-treated cells versus untreated N cells; °°$p<0.01$,°°°$p<0.001$: mQ-treated cells versus respective H, H/N, H/N/H untreated cells.

Supplemental Figure S10

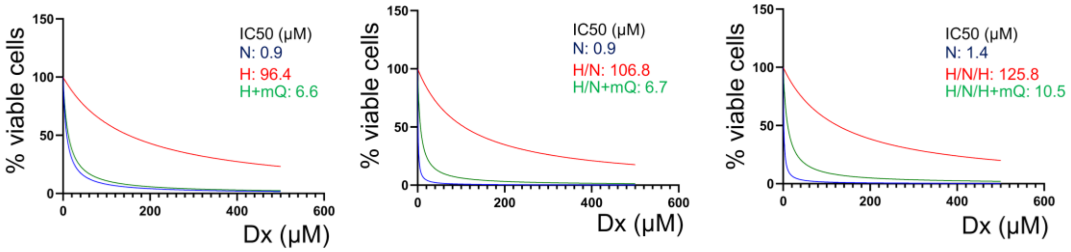

Supplemental Figure S10. Scavenging mitochondrial ROS sensitizes non-small cell lung cancer cells to docetaxel

NCI-H2228 cells were cultured in the following conditions: normoxia (at 20% O_2 for 24h, N), hypoxia (at 1% O_2 for 24h, H), hypoxia/normoxia (12h at 1% O_2 followed by 12h at 20% O_2, H/N), hypoxia/rnormoxia/hypoxia or intermittent hypoxia (12h at 1% O_2 followed by 12h at 20% O_2 and 12h at 1% O_2, H/N/H). After these incubation times, cells were treated for 48h in normoxia (20% O_2) with increasing concentrations (from 1 10^{-9} to 1×10^{-5}M) of docetaxel (Dx). When indicated, 0.4μM mQ was added. Cell viability was measured by a chemiluminescence-based assay, in technical quadruplicates (n=3 biological replicates). Representative (inhibitor) vs. normalized dose-response curves and relative IC_{50}, obtained with the GraphPad Prism 9 software.

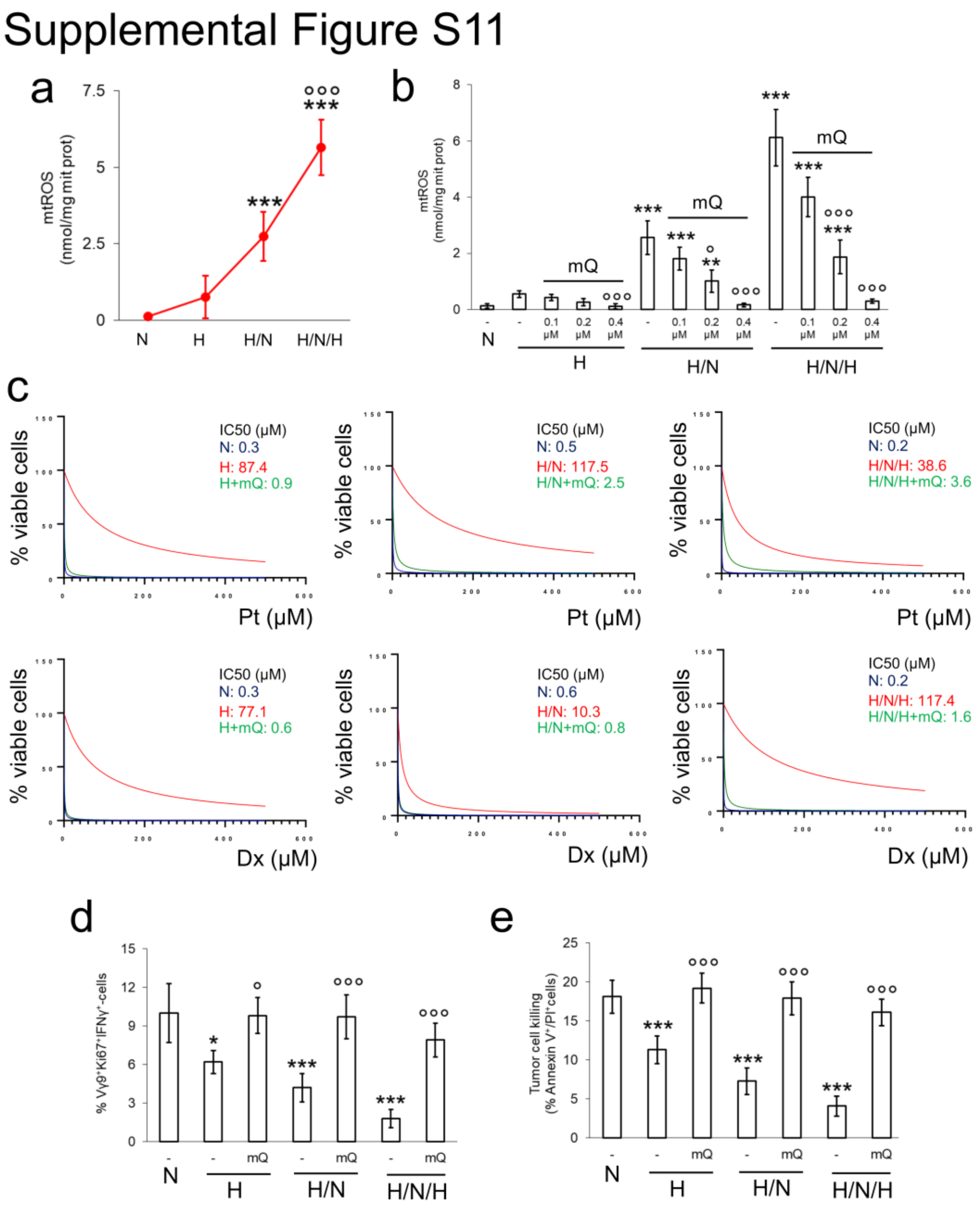

Supplemental Figure S11. Chemo-immuno-sensitizing effects of mitoquinol in NCI-H1563 cells grown in intermittent hypoxia

NCI-H1563 cells were cultured in the following conditions: normoxia (at 20% O_2 for 24h, N), hypoxia (at 1% O_2 for 24h, H), hypoxia/normoxia (12h at 1% O_2 followed by 12h at 20% O_2, H/N), hypoxia/normoxia/hypoxia or intermittent hypoxia (12h at 1% O_2 followed by 12h at 20% O_2 and 12h at 1% O_2, H/N/H). **a.** Mitochondrial ROS, measured spectrofluorimetrically, in technical triplicates.

Data are means±SD (n=4 biological replicates). ***p<0.001: H, H/N/H versus N cells; °°°p<0.001: H/N cells versus H cells. **b.** Hypoxic cells were treated with or without (-) 0.1, 0.2, 0.4μM mitoquinol (mQ). Normoxic cells were included as internal control. Mitochondrial ROS were measured spectrofluorimetrically, in technical quadruplicates. Data are means±SD (n=3 biological replicates). **p<0.01,***p<0.001:H, H/N, H/N/H cells or mQ-treated cells versus untreated N cells; °p<0.05,°°°p<0.001 mQ-treated cells versus respective H, H/N, H/N/H untreated cells. **c.** After normoxic and hypoxic cultures, cells were treated for 48h in normoxia (20% O_2) with increasing concentrations (from 1×10^{-9} to 1×10^{-5}M) of cisplatin (Pt, upper panels) and docetaxel (Dx, lower panels). When indicated, 0.4μM mQ was added. Cell viability was measured by a chemiluminescence-based assay, in technical quadruplicates (n=3 biological replicates). Representative [inhibitor] vs. normalized dose-response curves and relative IC_{50}, obtained with the GraphPad Prism 9 software. **d.** Percentage of $Ki67^{+}IFN\text{-}\gamma^{+}$ Vγ9Vδ2 T cells collected after the co-cultures with the NCI-H1563 cells, evaluated by flow cytometry, in technical duplicates. Data are means±SD (n=5 biological replicates). *p<0.05,***p<0.001: H, H/N, H/N/H versus N cells; °p<0.05,°°°p<0.001 mQ-treated cells versus respective H, H/N, H/N/H untreated cells. **e.** Percentage of annexin $V^{+}PI^{+}$ NCI-H1563 cells, as index of tumor cells killed by Vγ9Vδ2 T-cells, evaluated by flow cytometry, in technical duplicates. Data are means±SD (n=5 biological replicates). ***p<0.001: H, H/N, H/N/H versus N cells; °°°p<0.001 mQ-treated cells versus respective H, H/N, H/N/H untreated cells.

Supplemental Figure S12

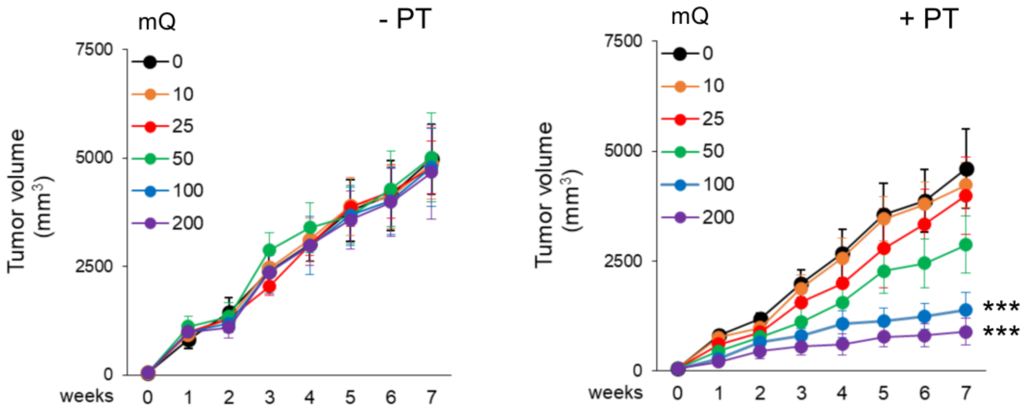

Supplemental Figure S12. Dose-response effects of cisplatin and mitoquinol combination in LAP-overexpression NCI-H2228 tumors

1×10^6 C/EBP-β LAP-overexpressing cells were injected subcutaneously in Hu-CD34$^+$NSG mice. When tumor reached the volume of 50 mm^3, animals (n=4/group) were randomized and treated for 6 weeks as it follows: vehicle (0) group, treated with 0.1ml saline solution intravenously (i.v.), once a week; mitoquinol (mQ) groups, treated with 10, 25, 50, 100 or 200 mg/kg daily via oral gavage, in the absence (-) or in the presence (+) of 2mg/kg cisplatin (PT) i.v., once a week. To induce LAP intratumourally, 1mg/ml doxycycline was added daily to the drinking water. Tumor growth was monitored by caliper. ***p<0.001: PT+mQ-groups vs. PT-group (weeks 2-7).

Supplemental Figure S13

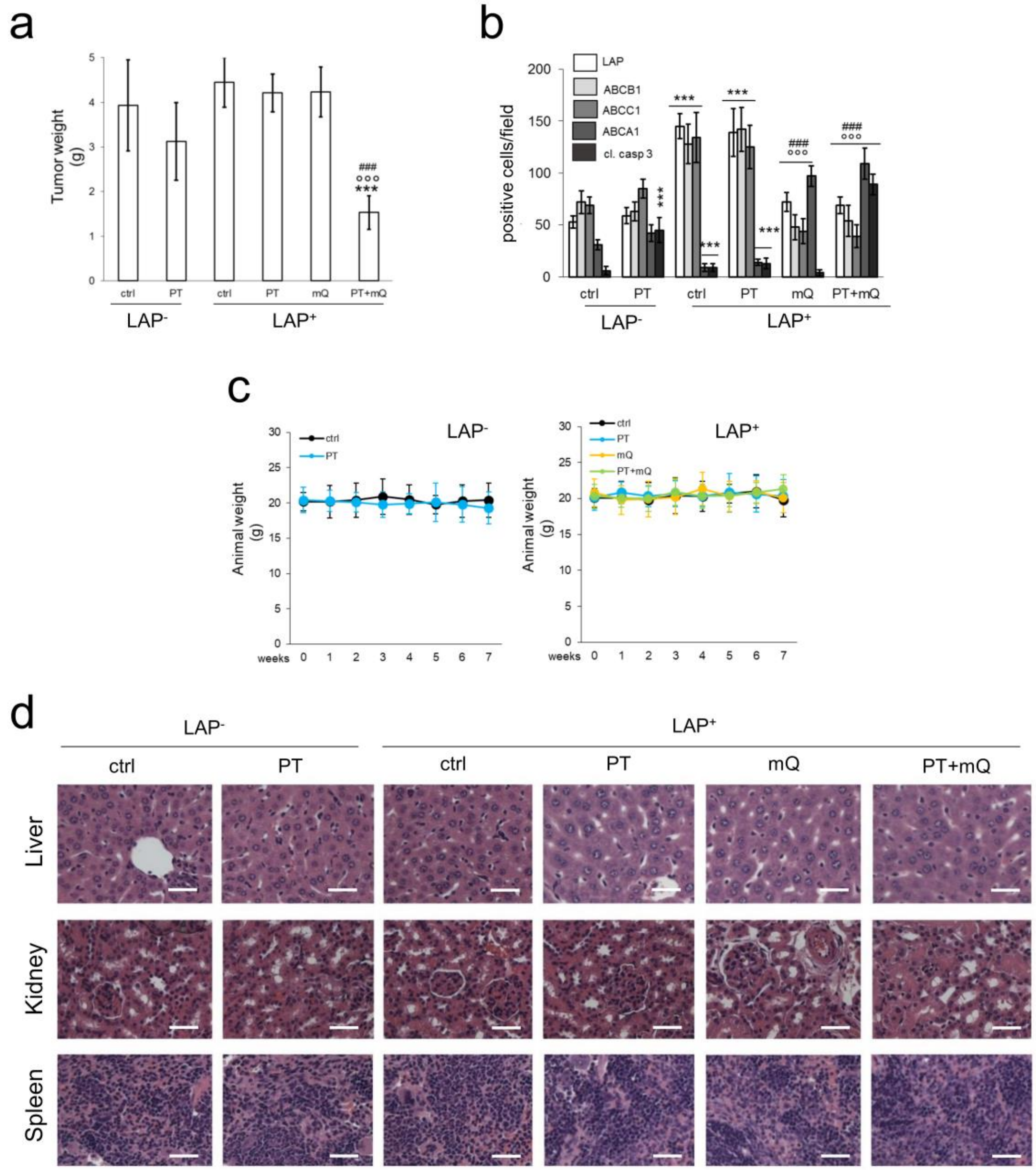

Supplemental Figure S13. Tumors and post-mortem tissues characterization

1×10^6 C/EBP-β LAP-overexpressing cells were injected subcutaneously in Hu-CD34$^+$NSG mice. When tumor reached the volume of 50 mm^3, animals (n=6/group) were randomized and treated for 5 weeks as it follows: 1) vehicle (ctrl) group, treated with 0.1 ml saline solution intravenously (i.v.), once a week; 2) cisplatin (PT) group, treated with 2 mg/kg cisplatin i.v., once a week; 3) mitoquinol (mQ)

group, treated with 100 µg/kg daily via oral gavage; 4) cisplatin + mitoquinol (PT+mQ) group, treated with 2 mg/kg cisplatin i.v., once a week and 100 µg/kg mitoquinol daily via oral gavage. To induce LAP intratumourally, 1 mg/ml doxycycline was added daily to the drinking water (LAP^+ mice). **a.** Weight of the excised tumors. Data are means±SD (n=6 tumors). ***p<0.001:LAP^+PT+mQ-group vs. LAP^-ctrl-group; °°°p<0.001:LAP^+PT+mQ-group vs LAP^+ctrl-group; ###p<0.001:LAP^+PT+mQ-group vs LAP^+PT-group. **b.** Immunohistochemical analysis quantification. The amount of cells positive for C/EBP-β LAP, ABCB1, ABCB1, ABCA1 and cleaved caspase 3 was calculated counting 200±25 cells/field, analyzing 5 fields for each treatment group, derived from each tumor and using Photoshop program. ***p<0.001:LAP^-PT-group vs LAP^-ctrl-group, LAP^+ctrl/PT-group vs. LAP^-ctrl-group; °°°p<0.001: LAP^+mQ/PT+mQ-group vs LAP^+ctrl-group; ###p<0.001: LAP^+mQ/PT+mQ-group vs LAP^+PT-group. **c.** Animals weight were monitored weekly. **d.** Representative hematoxylin-eosin staining of liver, kidneys and spleen examined *post-mortem*. For each experimental group a minimum of 5 field were examined. Liver, kidneys: 63× objective, 20× ocular, bar: 50 µm; spleen: 63× objective, 10× ocular, bar: 100 µm.

Supplemental Table S4. Hematochemical parameters of treated animals

**3.5 weeks**	**ctrl**	**PT**	**LAP ctrl**	**LAP PT**	**LAP mQ**	**LAP PT+mQ**
**RBC (x 10^6/µl)**	6.16 ± 1.34	5.93 ± 2.11	6.12 ± 1.76	5.12 ± 3.21	5.49 ± 0.929	5.98 ± 2.34
**Hb (g/dl)**	12.65 ± 1.43	12.34 ± 2.17	13.05 ± 0.45	12.14 ± 2.393	12.45 ± 2.39	13.08 ± 1.98
**WBC (x 10^3/µl)**	16.34 ± 1.23	16.98 ± 4.32	15.43 ± 2.36	17.05 ± 2.09	17.34 ± 2.18	17.23 ± 3.04
**PLT (x 10^3/µl)**	876 ± 237	723± 265	709 ± 213	765± 394	807 ± 209	783± 109
**LDH (U/l)**	6574 ± 801	7098 ± 802	6723 ± 507	6732 ± 409	6574 ± 403	6092 ± 302
**AST (U/l)**	176 ± 34	202 ± 44	182 ± 42	145 ± 45	167 ± 39	134 ± 44
**ALT (U/l)**	43 ± 1	35 ± 21	44 ± 14	35± 10	45 ± 28	45± 9
**AP (U/l)**	103 ± 21	114 ± 27	117 ± 23	103 ± 21	129 ± 41	103 ± 3
**Creatinine (mg/l)**	0.064 ± 0.011	0.087 ± 0.008	0.067 ± 0.009	0.065 ± 0.010	0.066 ± 0.011	0.064 ± 0.009
**CPK (U/l)**	231 ± 47	198 ± 34	204 ± 39	234 ± 45	241 ± 56	234 ± 12

**7 weeks**	**ctrl**	**PT**	**LAP ctrl**	**LAP PT**	**LAP mQ**	**LAP PT+mQ**
**RBC (x 10^6/µl)**	4.34 ± 1.16	3.98 ± 0.84	4.39 ± 1.13	2.45 ± 1.02	3.49 ± 0.67	3.12 ± 1.03
**Hb (g/dl)**	11.14 ± 0.87	10.21 ± 0.94	11.98 ± 0.83	10.23 ± 0.87	11.92 ± 0.64	10.92 ± 0.87
**WBC (x 10^3/µl)**	15.17 ± 3.42	11.98 ± 2.18	13.98 ± 2.17	11.82± 0.93	12.38 ± 1.26	12.19 ± 0.76
**PLT (x 10^3/µl)**	893 ± 189	567 ± 98*	809 ± 182	604 ± 93	678 ± 182	598 ± 117
**LDH (U/l)**	9834 ± 348	10921 ± 298	101231 ± 509	9845 ± 807	7832 ± 903	7093 ± 409
**AST (U/l)**	154 ± 39	178 ± 28	189± 45	176 ± 28	201 ± 65	209 ± 78
**ALT (U/l)**	45 ± 12	46 ± 17	37±18	45 ± 11	43 ± 14	43 ± 14
**AP (U/l)**	109 ± 29	102 ± 15	87 ± 54	104 ± 33	96 ± 35	89 ± 17
**Creatinine (mg/l)**	0.065 ± 0.009	0.099 ± 0.009*	0.064 ± 0.007	0.087 ± 0.005*	0.071± 0.007	0.078 ± 0.011
**CPK (U/l)**	234 ± 57	198 ± 76	236 ± 52	209 ± 45	231 ± 71	201 ± 74

Mice (n=6) were treated as described the Figure 7. Blood was collected at week 3.5 and at week 7, immediately after euthanasia, and analyzed for red blood cells (RBC) count, hemoglobin (Hb), white blood cells (WBC) count, platelets (PLT) count, lactate dehydrogenase (LDH), aspartate aminotransferase (AST), alanine aminotransferase (ALT), alkaline phosphatase (AP), creatinine, creatine phosphokinase (CPK). Data are presented as means ± SD. *$p<0.05$: vs ctrl
